# The human milk component *myo*-inositol promotes neuronal connectivity

**DOI:** 10.1073/pnas.2221413120

**Published:** 2023-07-11

**Authors:** Andrew F. Paquette, Beatrice E. Carbone, Seth Vogel, Erica Israel, Sarah D. Maria, Nikita P. Patil, Saroj Sah, Dhrubajyoti Chowdhury, Ilona Kondratiuk, Beau Labhart, Ardythe L. Morrow, Shay C. Phillips, Chenzhong Kuang, Dirk Hondmann, Neeraj Pandey, Thomas Biederer

**Affiliations:** ^a^Department of Neurology, Yale School of Medicine, New Haven, CT 06511; ^b^Department of Neuroscience, Tufts University School of Medicine, Boston, MA 02111; ^c^United States Department of Agriculture Human Nutrition Research Center on Aging, Boston, MA 02111; ^d^Global Analytical Sciences, Reckitt/Mead Johnson Nutrition, Evansville, IN 47712; ^e^Perinatal Institute, Cincinnati Children’s Hospital Medical Center, Cincinnati, Cincinnati, OH 45229; ^f^Global Discovery, Mead Johnson Nutrition, Evansville, IN 47712; ^g^Medical and Scientific Affairs, Reckitt, Slough SL1 3UH, United Kingdom

**Keywords:** synapse formation, brain development, nutritional neuroscience, *myo*-inositol, DHA

## Abstract

Effects of micronutrients on brain connectivity are incompletely understood. Analyzing human milk samples across global populations, we identified the carbocyclic sugar *myo*-inositol as a component that promotes brain development. We determined that it is most abundant in human milk during early lactation when neuronal connections rapidly form in the infant brain. *Myo*-inositol promoted synapse abundance in human excitatory neurons as well as cultured rat neurons and acted in a dose-dependent manner. Mechanistically, *myo*-inositol enhanced the ability of neurons to respond to transsynaptic interactions that induce synapses. Effects of *myo*-inositol in the developing brain were tested in mice, and its dietary supplementation enlarged excitatory postsynaptic sites in the maturing cortex. Utilizing an organotypic slice culture system, we additionally determined that *myo*-inositol is bioactive in mature brain tissue, and treatment of organotypic slices with this carbocyclic sugar increased the number and size of postsynaptic specializations and excitatory synapse density. This study advances our understanding of the impact of human milk on the infant brain and identifies *myo*-inositol as a breast milk component that promotes the formation of neuronal connections.

## Significance

The roles of micronutrients and bioactive dietary compounds in neuronal wiring are only beginning to be addressed. This study reveals substantial benefits of the human milk component *myo*-inositol for developing synapses across species, including in human neurons. These findings demonstrate that *myo*-inositol promotes neuronal connectivity and can guide dietary recommendations across life stages. This can be significant for pediatric nutrition and the improvement of infant formulas in underresourced areas with conditions that prevent sufficient breastfeeding. Moreover, this carbocyclic sugar can promote synapse density in mature brain tissue.

The formation and maintenance of brain connectivity are guided by an interplay of genetics, experience, and environment. The impact of these factors can be considered particularly important at two stages of life, when synaptic connections rapidly form in the developing brain and when synapses are gradually lost in aging (1, 2). Diet is one environmental factor, yet the effects of bioactive dietary compounds on the formation and maintenance of neuronal connectivity remain to be defined. In early postnatal development, breast milk is rich in micronutrients and bioactive compounds that could support brain development. Indeed, this complex and dynamic fluid offers short- and long-term health benefits to infants, including increased performance in cognitive tasks (3–5). Further, observational studies on the other side of the lifespan support that dietary factors can be associated with healthy brain aging (6, 7).

These results raise the question of how diet can impact brain connectivity. Postnatal brain development may be particularly sensitive to dietary factors due to the relative permeability of the gut and the blood–brain barrier for small molecules in infants (8). Aiming to identify compounds that are bioactive in brain development, we regarded breastmilk as a rich potential source given its complexity and benefits for infants. We considered breastmilk components with temporal profiles over lactation that match stages of infant brain development to be of particular interest. Moreover, compounds exhibiting the same profile over lactation independent of geography and racial diversity could play important general roles in the mother–baby dyad relationship.

To address these questions, we profiled human milk samples collected across global sites. This identified the carbocyclic sugar *myo*-inositol (*cis*-1,2,3,5-*trans*-4,6-cyclohexanehexol) as a component that is most prominent during the early stages of lactation when neuronal connections form rapidly in the infant brain. *Myo*-inositol is provided in the Western diet of an adult at approximately 1 g per day and is additionally produced in the brain and other organs (9). It exists in the brain both in its free form, which can cross the blood–brain barrier (10), and as metabolized derivatives that participate in a range of cellular processes including second messenger signaling by phosphatidylinositol polyphosphates (11–13). Using a complementary set of models––primary rodent neurons, human neurons, and mice––we determined that *myo*-inositol has synaptogenic effects in postnatal development. Moreover, treatment of mature organotypic slice cultures prepared from mice elevated synaptic abundance and size. These results identify *myo*-inositol as a bioactive dietary compound that promotes neuronal connectivity. Our findings advance insights into how human milk supports infant brain development and the roles of dietary factors in brain connectivity.

## Results

### *Myo*-Inositol Is a Dynamic Component of Human Milk That Peaks Early in Lactation.

We collected milk from mothers at three global sites in Mexico City, Shanghai, and Cincinnati over 1 y of lactation in the Global Exploration of Human Milk (GEHM) study, using controlled protocols described previously (14, 15). Demographic data are shown in *SI Appendix*, Table S1. These longitudinal samples allow for determining the global maternal provisioning of human milk factors in the first y of infant life.

**Fig. 1.**
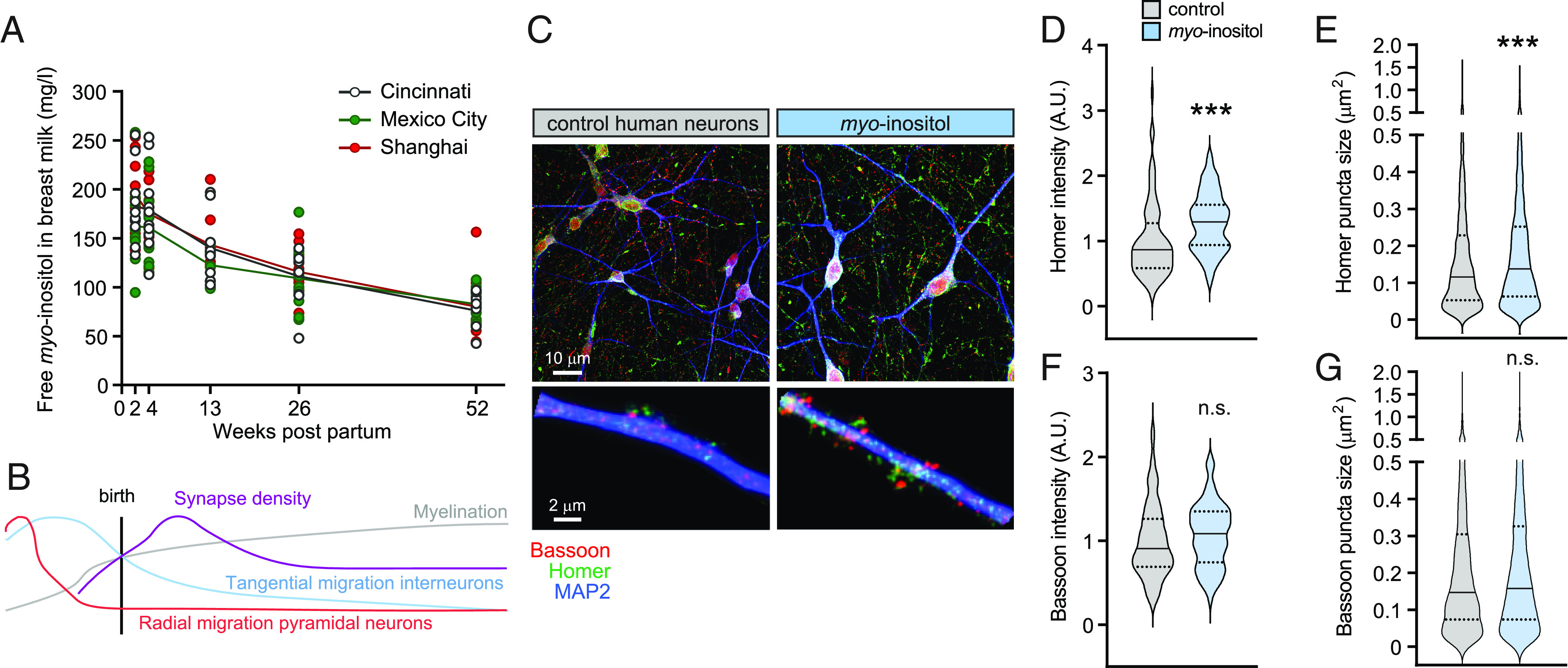
*Myo*-inositol peaks in human milk during early infant brain development and this carbocyclic sugar promotes the abundance and size of excitatory postsynaptic sites in human glutamatergic neurons. (*A*) Free *myo*-inositol content in human milk is highest in the first wk of lactation. Samples were longitudinally collected from ten mothers per site in Shanghai, Mexico City, and Cincinnati over 52 wk. Circles, *myo*-inositol concentration per mother. Lines, mean concentration per geography. (*B*) Temporal profiles of processes underlying cortical developmental. (*C*, *Top*), representative confocal images of human glutamatergic–enriched cortical neurons 24 d after plating, cultured under control conditions (*C*, *Left*) or with *myo*-inositol at 2 mM (*C*, *Right*). Immunostainings were performed for presynaptic Bassoon (red), excitatory postsynaptic Homer (green), and dendritic MAP2 (blue). (*C*, *Bottom*) enlarged dendritic segments. (*D* and *E*) Quantification of images as in *C* determined that *myo*-inositol increases in human glutamatergic neurons the abundance of postsynaptic Homer measured as immunostaining intensity per dendritic area (*D*) and the size of Homer-positive specializations (*E*). Violin plots show data distribution. Solid lines mark the median and dotted lines the quartiles. Asterisks show statistical differences of mean values, which are not plotted. (Student’s *t* test, two-tailed unpaired; dendritic segments from N = 86 control/71 *myo*-inositol-treated neurons) ***P < 0.001. (*F* and *G*) *Myo*-inositol treatment did not significantly increase presynaptic Bassoon abundance (*F*) and size of Bassoon-positive sites (*G*). Violin plots show data as in *D* and *E*. n.s., not significant.

We focused on components that are more abundant in human than cow milk and analyzed the carbocyclic sugar inositol, a polyol with six hydroxyl groups (16). *Myo*-inositol is the most common epimer and is found in tissues with high glucose utilization, such as the brain (17). Inositol is present in human milk in the free *myo*-inositol form and bound forms such as inositol phosphate and phosphatidylinositol, and across global samples, 93% of human milk inositol was quantified in the form of free *myo*-inositol (*SI Appendix*, Table S2). Analysis of the GEHM samples determined that free *myo*-inositol content was consistently high postpartum, with a global mean of 180 ± 41 µg/mL at 2 wk (Fig. 1*A* and *SI Appendix*, Table S2). Free *myo*-inositol intake by infants at 2 wk was estimated as 105 ± 24 mg/day (mean ± SD) (*SI Appendix*, Table S2). *Myo*-inositol in human milk subsequently decreased, which was most pronounced after early lactation (Fig. 1*A*). The *myo*-inositol profile across the study geographies was indistinguishable, with no difference by country (*P* = 0.35) (*SI Appendix*, Table S3). Like free inositol, total inositol concentration decreased over lactation (*P* < 0.01) with no significant interaction effect of maternal country (*P* = 0.36) (*SI Appendix*, Table S4).

### *Myo*-Inositol Promotes in Human Neurons the Abundance of Specializations Positive for the Excitatory Postsynaptic Marker Homer.

The profile of inositol in human milk tracked the peak of synaptogenesis in the infant cortex, a crucial process of postnatal development (1, 2) (Fig. 1*B*; see also *SI Appendix*, Supplemental Results). Thus, we aimed to test whether *myo*-inositol is a human milk compound impacting neuronal connectivity. We analyzed this in human glutamatergic–enriched cortical neurons derived from induced pluripotent stem cells. Immunostaining for the presynaptic active zone marker Bassoon and the excitatory postsynaptic scaffold protein Homer showed that synaptic specializations were formed by 21 d after plating (Fig. 1*C*). We first determined to what extent synaptogenic effects of bioactive compounds identified in rodent neurons can be replicated in human neurons. This validated that the ω-3 fatty acid docosahexaenoic acid (DHA) promoted the number of Homer-positive sites in human neurons similar to its previously described effects in rat neurons (18, 19) (*SI Appendix*, Fig. S1). Human neurons were treated with 2 mM *myo*-inositol 3 d after plating, when neurites started to appear. Elevating *myo*-inositol did not alter the dendritic complexity and length of human glutamatergic neurons, supporting the proper health of treated cultures (*SI Appendix*, Fig. S2). Analysis of excitatory Homer-positive sites determined that *myo*-inositol treatment increased their abundance by 29 ± 8 % in human neurons as assessed by immunostaining intensity (Student’s *t* test; *P* = 0.0007) (Fig. 1*D*). It also enlarged Homer-positive puncta modestly but significantly by 13 ± 2.5 % (Student’s *t* test; *P* < 0.0001) (Fig. 1*E*). We did not observe significant effects of *myo*-inositol treatment on presynaptic sites when measuring Bassoon abundance or puncta size (Fig. 1 *F* and *G*). Colocalization of Homer and Bassoon puncta could not be assessed due to the dense innervation and high extent of staining. These results provided evidence that *myo*-inositol promotes the development of Homer-positive specializations along dendrites of human neurons.

### Synapse Formation Is Promoted by *Myo*-Inositol in a Dose-Dependent Manner.

**Fig. 2.**
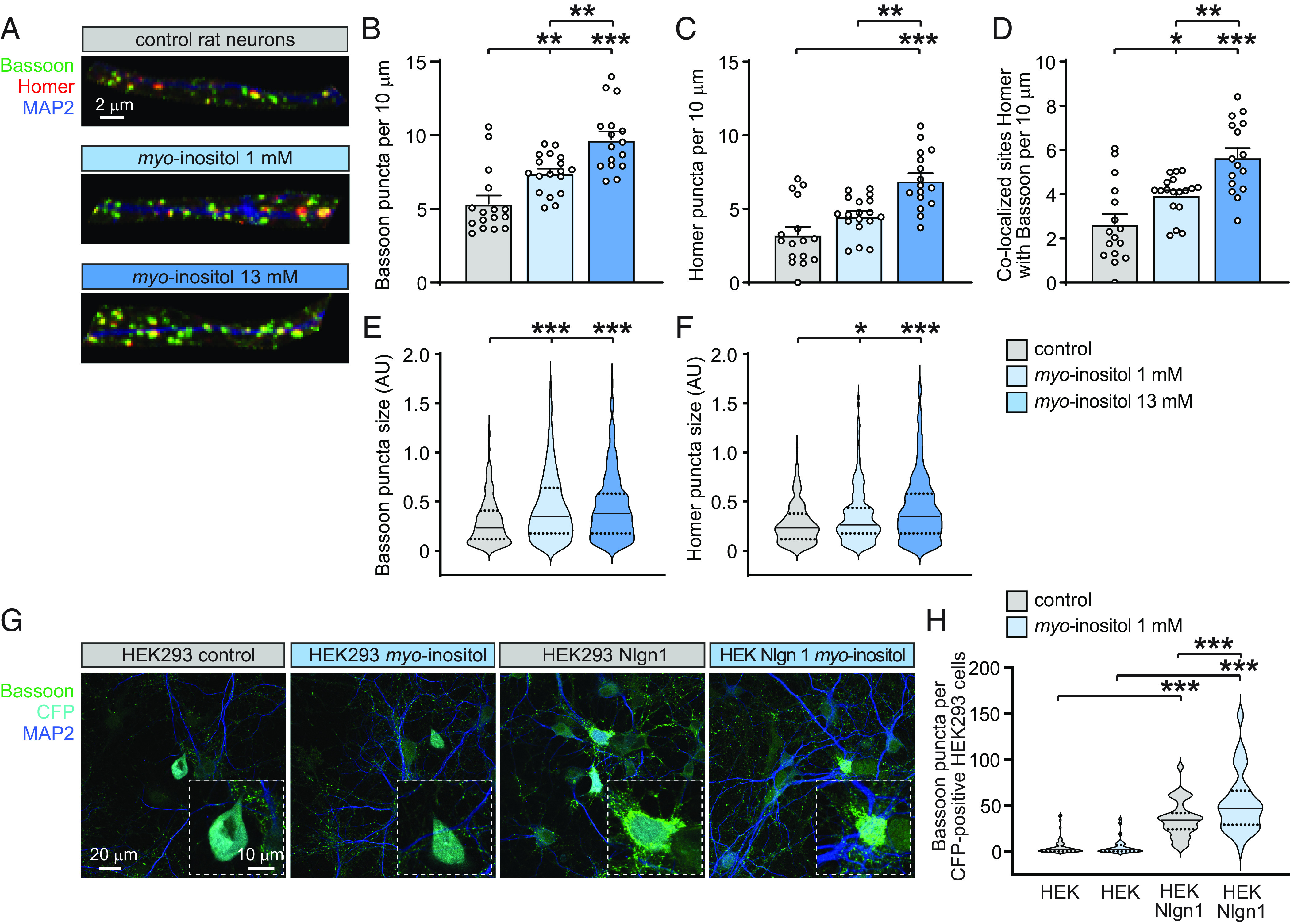
Synapse size and number are increased by *myo*-inositol in a dose-dependent manner, and it promotes neuronal responsiveness to synaptogenic Neuroligin 1. (*A*) Representative confocal images of dendritic segments of control primary rat neurons at 14 div (*A*, *Top*), or of neurons treated with *myo*-inositol at 1 mM (*A*, *Center*) or 13 mM (*A*, *Bottom*). Immunostainings show presynaptic Bassoon (green), postsynaptic Homer (red), and dendritic MAP2 (blue). (*B*–*F*) Quantification of images as in *A* determined that *myo*-inositol increases in a dose-dependent manner the density of Bassoon (*B*) and Homer (*C*) puncta and of the synaptic sites where they colocalize (*D*). *Myo*-inositol also increased the size of Bassoon and Homer puncta (*E* and *F*), with data distribution shown in Violin plots as in Fig. 1 (one-way ANOVA with Tukey’s multiple comparison test; N = 16 to 18 dendritic segments per condition). (*G*) Confocal images of rat hippocampal neurons cultured in the presence of vehicle control or *myo*-inositol at 1 mM as indicated. Neurons were cocultured with HEK293-expressing CFP as transfection marker (cyan) that served as negative control, or with HEK293 cells coexpressing CFP and Neuroligin 1. At 14 div., immunostaining was performed for the presynaptic active zone marker Bassoon (green) and the dendritic marker MAP2 (blue). Insets show enlarged cocultured HEK293 cells. (*H*) Quantification of images as in *G* showed that contact with HEK293 cells expressing Neuroligin 1 induced neurons to assemble Bassoon-positive specializations, as expected. *Myo*-inositol treatment increases this synaptogenic response by a further 50 ± 13 %. Violin plots show data distribution (one-way ANOVA with Tukey’s multiple comparison test; N = 57 to 72 HEK293 cells per condition from three independent experiments).

We extended our analysis of *myo*-inositol to dissociated rat hippocampal neurons, which have been used previously to analyze the ω-3 fatty acid DHA as a micronutrient bioactive in synapse development (18, 19). Regular neuronal culture medium includes *myo*-inositol in low amounts. We first determined using a custom-made medium lacking *myo*-inositol that it is required for neurons to develop properly in vitro (div) (*SI Appendix*, Fig. S3). We next cultured neurons in regular medium or added *myo*-inositol starting at 4 div, when these primary neurons begin to differentiate in vitro. Synaptic analyses were performed at 14 div by immunostaining for the presynaptic active zone protein Bassoon and the excitatory postsynaptic Homer, together with staining for MAP2 to trace dendrites, followed by confocal microscopy (Fig. 2*A*). We validated the Homer antibody for labeling excitatory postsynaptic sites by determining that the puncta it detected colocalized to the same extent with Bassoon as the excitatory postsynaptic marker PSD-95 and that 80 ± 3 % of PSD-95 puncta were positive for Homer (*SI Appendix*, Fig. S4). Quantitative analysis determined that the density of Bassoon-positive presynaptic sites along dendrites was increased by 38 ± 11 % (Student’s *t* test; *P* = 0.002) upon treatment with 1 mM *myo*-inositol compared to vehicle-treated control neurons (Fig. 2*B*). The number of Homer-labeled postsynaptic sites was similarly elevated in the presence of 1 mM *myo*-inositol by 40 ± 19 % (Student’s *t* test; *P* = 0.039) (Fig. 2*C*). Synapses are defined by the apposition of pre- and post-synaptic sites, and 1 mM *myo*-inositol strongly promoted the number of postsynaptic Homer sites colocalized with Bassoon by 50 ± 18 % (Student’s *t* test; *P* = 0.010) (Fig. 2*D*). With these effects, *myo*-inositol shared the synaptogenic effects of DHA (*SI Appendix*, Fig. S5) (18, 19). *Myo*-inositol acted in a dose-dependent manner and had even higher effects at 13 mM (Fig. 2 *B*–*D*). Moreover, treatment of neurons with this carbocyclic sugar enlarged Bassoon- and Homer-positive specializations (Fig. 2 *E* and *F*). We additionally compared *myo*-inositol effects on excitatory vs. inhibitory synaptic specializations, which determined that *myo*-inositol increased the density of VGLUT1 puncta along MAP2-positive dendrites by 26 ± 12 % compared to control-treated neurons (Student’s *t* test; *P* = 0.035), in agreement with its effect on the excitatory postsynaptic marker Homer (*SI Appendix*, Fig. S6 *A* and *B*). The number of VGAT-positive inhibitory presynaptic sites, which are less abundant, was also increased after *myo*-inositol addition by 35 ± 16 % compared to control neurons (Student’s *t* test; *P* = 0.034) (*SI Appendix*, Fig. S6*C*). These results demonstrated that *myo*-inositol strongly promoted the development of synaptic specializations in rat hippocampal neurons.

### Higher Responsiveness of Developing Neurons to Adhesive Synaptogenic Interactions upon Elevation of *Myo*-Inositol.

Transsynaptic interactions promote synapse development, and we tested whether *myo*-inositol treatment altered how neurons responded to the synaptogenic activity of the postsynaptic adhesion molecule neuroligin 1 (20). Utilizing HEK293 cells heterologously expressing neuroligin 1 that were cocultured with dissociated rat hippocampal neurons, we observed the expected induction of presynaptic sites positive for the marker Bassoon atop the Neuroligin 1–expressing HEK293 cells (Fig. 2 *G* and *H*). Administration of *myo*-inositol significantly enhanced the synaptogenic effect of Neuroligin 1. This carbocyclic sugar hence promoted the induction of presynaptic sites as measured in the coculture system.

### *Myo*-Inositol Enlarges Excitatory Synaptic Sites in the Cortex.

**Fig. 3.**
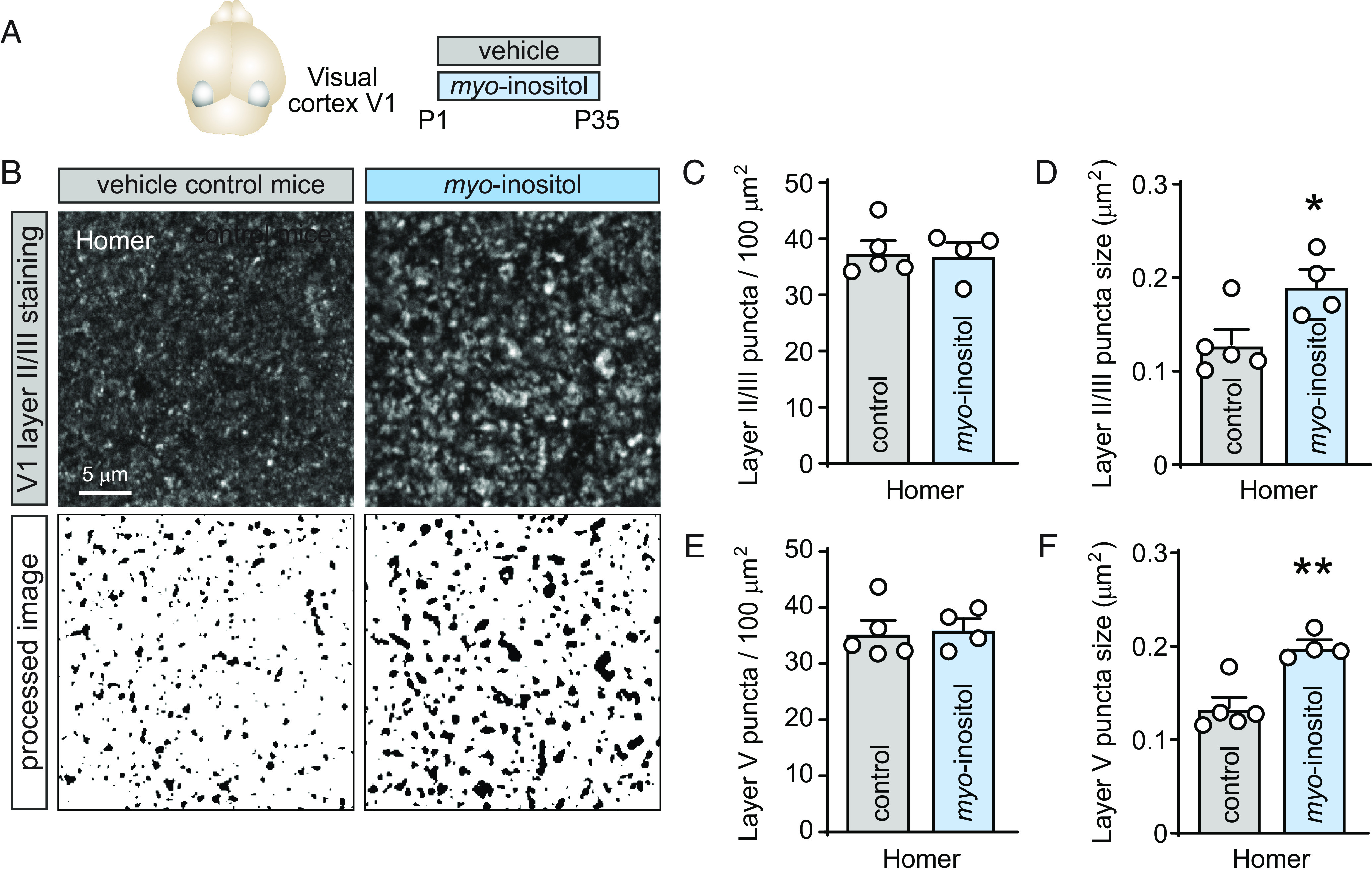
Cortical synapse size increases when *myo*-inositol is supplemented during development. (*A*) For analysis of the visual cortex area V1, the diet of mouse pups was supplemented from birth with *myo*-inositol until postnatal day 35. (*B*, *Top*), single optical confocal sections of synaptic Homer immunostaining in binocular primary visual cortex area V1b, layer II/III in control (*B*, *Left*), and *myo*-inositol-supplemented mice (*B*, *Right*) at postnatal day 35. Mice received 50 mg *myo*-inositol/kg body weight/day from birth. (*B*, *Bottom*), puncta masks after image processing. (*C* and *D*) Quantification in layer II/III of V1 from images as in *B* of synaptic Homer puncta density (*C*) and size (*D*) of control and *myo*-inositol-supplemented animals. Animal averages are shown, and means were compared (Student’s *t* test, two-tailed unpaired; control, N = 5 mice, data averaged from 3/2/2/2/2 sections per mouse; *myo*-inositol, N = 4, data averaged from 2/2/2/2 sections per mouse). (*E* and *F*) Quantification in layer V of V1b of synaptic Homer puncta density (*E*) and size (*F*) of mice treated as in *A* and imaged as in *B* (Student’s *t* test, two-tailed unpaired; control, N = 5 mice; *myo*-inositol, N = 4).

We next analyzed the effects of *myo*-inositol on synaptic connectivity in vivo and selected the cortex due to its relevance for higher brain functions. We chose the visual cortex V1 as it is well suited to study the postnatal development of neuronal connectivity, offering the advantage that cortical sensory areas undergo highly stereotyped development (21). We analyzed the binocular zone V1b where higher-order integration of visual signals occurs. Immunohistochemical stainings for synaptic specializations were performed at postnatal day 35 (P35), when V1b undergoes final maturation (21). Mice received dietary supplementation with 50 mg *myo*-inositol/kg body weight/day once a day orally, from birth until P35 to probe effects while the V1 is maturing (Fig. 3*A*). This amount was chosen based on the dietary uptake in infants (*SI Appendix*, Extended Methods). Body weight was unaffected (*SI Appendix*, Fig. S7). We first analyzed synaptic markers in layer II/III, which is rich in corticocortical connections. Validating that Homer immunostaining marks excitatory postsynaptic sites, we determined that 89 ± 2 % of Homer puncta colocalized with PSD-95 (*SI Appendix*, Fig. S8). No effect of dietary *myo*-inositol supplementation was found on the density of the analyzed excitatory and inhibitory synaptic markers (Fig. 3 *B* and *C* and *SI Appendix*, Fig. S9 and Supplemental Results). We extended our analysis to the size of synaptic specializations, a correlate of synaptic strength in the neocortex (22). Importantly, the size of Homer puncta in V1 was strongly increased in *myo*-inositol-supplemented mice by 49 ± 18%, from 0.129 ± 0.02 µm^2^ in control animals to 0.192 ± 0.02 µm^2^ in *myo*-inositol-supplemented mice (Fig. 3*D*) (Student’s *t* test; n = 5/4, t = 2.764, *P* = 0.028). Cumulative distribution frequency analysis of Homer puncta supported that they were enlarged by *myo*-inositol across their full size range (*SI Appendix*, Fig. S9). The size of other synaptic markers was unaffected (*SI Appendix*, Fig. S9).

We extended our analysis to layer V; we choose this layer because it contains multiple classes of commissural and corticofugal projection neurons distinct from the projection neurons in layers II/III. Quantification of puncta in layer V again determined that *myo*-inositol supplementation did not alter the density of Homer puncta but enlarged them (Fig. 3 *E* and *F*), with other excitatory and inhibitory synaptic markers unaltered (*SI Appendix*, Fig. S10). These results demonstrated that *myo*-inositol increased the size of Homer-positive excitatory postsynaptic sites in the maturing cortex.

### The Abundance of Excitatory Synaptic Sites in Mature Brain Tissue Is Promoted by Treatment with *Myo*-Inositol.

**Fig. 4.**
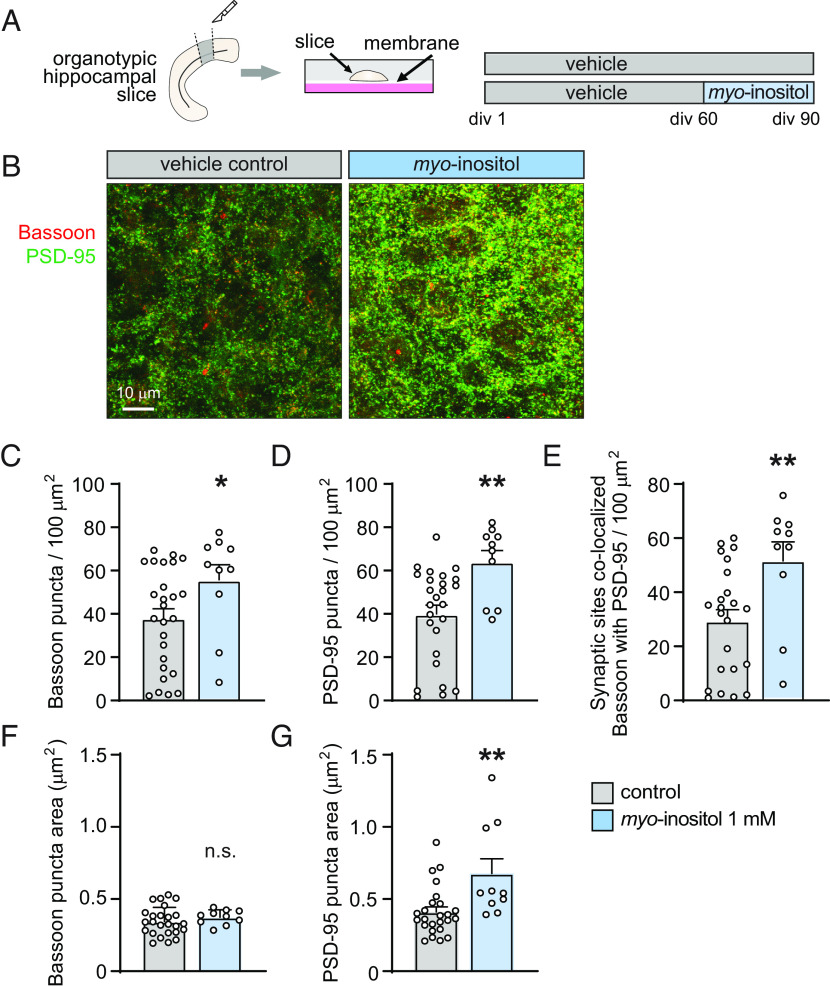
*Myo*-inositol is synaptogenic in mature hippocampal organotypic slice cultures. (*A*) Organotypic slice cultures were prepared from the hippocampus of mouse pups and cultured for 90 d. A subset of cultured slices was treated from 60 to 90 div with *myo*-inositol at 1 mM. (*B*) Representative confocal images of CA3 stratum lucidum of organotypic hippocampal slice cultures for 90 div under control conditions (*B*, *Left*) or after treatment with *myo*-inositol at 1 mM from 60 to 90 div (*B, Right*). Slice cultures were stained for presynaptic Bassoon (red) or the excitatory postsynaptic marker PSD-95 (green). (*C*–*E*) Quantification of immunostainings from the hippocampal CA3 area as in *B* showed that *myo*-inositol increases the density of presynaptic Bassoon (*C*) and postsynaptic PSD-95-positive specializations (*D*) and of sites where these pre- and post-synaptic markers colocalize (*E*). (*F* and *G*) Quantification of images as in *B* determined that *myo*-inositol does not change the size of Bassoon-positive sites (*F*) but enlarges PSD-95-positive specializations (*G*) in the hippocampal CA3 area (*C*–*G*, one-way ANOVA with Tukey’s multiple comparison test; N = 25 control and N = 10 *myo*-inositol slices from two independent experiments).

Finally, we asked whether *myo*-inositol is not only bioactive during development but also in mature brain tissue. We tested this in interface organotypic slice cultures prepared from the hippocampus of neonate mice, a preparation in which the structural and synaptic organization of the original tissue is preserved (23). Organotypic slices can be cultured for prolonged periods of time ex vivo, and we selected them as a system because they allow precise control over treatment conditions to obtain results complementary to our in vitro studies. They reach maximal functional connectivity by 3 wk in culture, similar to the time course in vivo, and acquire by the third wk in culture the complex morphological appearance of dendritic spines found in the mature brain (24, 25). We therefore considered that these slice cultures reached maturity by 60 d in culture. At this time point, we started treating them for 30 d with *myo*-inositol at 1 mM, or vehicle control (Fig. 4*A*). At the end of the treatment period, slice cultures were subjected to immunohistochemical staining to measure the abundance and density of presynaptic Bassoon and the postsynaptic excitatory marker PSD-95 (Fig. 4*B*). We analyzed the CA3 area as it is among the brain regions that show the most pronounced differential gene expression during aging, including genes involved in synaptic signaling and cell communication (26). This area may hence be particularly responsive to synaptogenic interventions. Quantification of synaptic immunostainings in the CA3 stratum lucidum showed that *myo*-inositol addition to mature organotypic hippocampal slices increased presynaptic Bassoon puncta density by 46 ± 22 % (Student’s *t* test; *P* = 0.048) and promoted the density of postsynaptic PSD-95 puncta by 61 ± 19 % (Student’s *t* test; *P* = 0.004) (Fig. 4 *C* and *D*). This corresponded with a striking 3.1-fold increase in synaptic sites where both markers colocalized (Student’s *t* test; *P* < 0.0001) (Fig. 4*E*). Moreover, *myo*-inositol addition to the cultured slices enlarged PSD-95-positive specializations, while the size of presynaptic Bassoon puncta remained unchanged (Fig. 4 *F* and *G*). The use of organotypic slice cultures provided the opportunity of additionally studying possible acute effects on microglia, the brain-resident immune cells which play roles in synapse formation and turnover during development (27). Immunostaining with the microglial marker Iba1 found that its staining intensity decreased in mature organotypic slice cultures after they were treated with *myo*-inositol (*SI Appendix*, Fig. S11). This is indicative of lower microglia activity which may reduce synapse elimination and complement synaptogenic effects of *myo*-inositol on neurons. Together, these results showed that *myo*-inositol treatment elevated synapse number when added to mature brain tissue.

## Discussion

In the developing brain, synapse formation occurs in conjunction with the refinement of synaptic connectivity (20, 28, 29) to ensure proper wiring. Effects of bioactive compounds from outside the brain on these processes are being investigated for microbiome factors but not well understood for dietary components. We report here that *myo*-inositol is prominent in human milk and highest during the early stages of infant brain development. Results from multiple models support that it robustly promotes synaptic connectivity. *Myo*-inositol increased the size and density of synaptic specialization in cultured primary rat neurons, and this effect was conserved in human glutamatergic neurons. Dietary *myo*-inositol also enlarged excitatory postsynaptic specializations in the cortex of mice. These effects were not limited to brain development and treatment of mature organotypic slice cultures prepared from mouse hippocampus increased synapse density and postsynaptic size.

On a mechanistic level, our studies of cultured neurons show that *myo*-inositol strongly promoted the size and number of excitatory postsynaptic sites, acts in a dose-dependent manner, and its effects are conserved across species. Our analysis of sites where pre- and post-synaptic markers colocalize shows that in cultured rat neurons and organotypic slice cultures, *myo*-inositol also promotes the density of synapses. The molecular pathways that are impacted by *myo*-inositol to promote synaptic connectivity remain to be determined. *Myo*-inositol improves synapse development in human neuronal cultures in which glia are absent, which supports that its effects on the assembly of synaptic sites are, at least in part, cell autonomous. Our result that *myo*-inositol modulates the responsiveness of neurons to the synaptogenic adhesion molecule Neuroligin 1 in a reduced system provides evidence that this carbocyclic sugar could increase the ability of neurons to assemble presynaptic sites. Here, increased *myo*-inositol may elevate synaptogenic signaling through inositol polyphosphate second messenger pathways that modulate cellular trafficking, among other processes (13). A link of *myo*-inositol metabolism to mitochondrial function has also been proposed (30), which could impact synaptogenic processes. Our application of organotypic slice cultures as a more intact ex vivo model can enable future mechanistic studies, which can then be extended to animals. Because *myo*-inositol is prominent in astrocytes (11) and our data together with others (31) indicate a correlation with microglial activity, the contributions of glia cells to *myo*-inositol effects in intact tissue need to be considered.

The fact that the size of excitatory postsynaptic sites positive for Homer but not their density was increased in the cortex of *myo*-inositol supplemented mice may be due to regulatory mechanisms balancing synapse formation in vivo, which may be lacking in reduced culture systems. As synapse size predicts transmission strength in the cortex (22), these larger excitatory synaptic sites upon elevated *myo*-inositol could make connectivity more robust even when synapse numbers are staying unchanged. While no apparent effects on presynaptic sites were observed in vivo, future studies can analyze a possible more subtle impact on their organization as well as on the coordination of pre- and post-synaptic growth, which usually occurs in concert. With respect to the effects of *myo*-inositol during the lifetime of synapses, it can additionally be addressed to what extent *myo*-inositol may first promote the number of immature synapses and then their maturation. Further, comparative analyses of its effects across brain regions can elucidate neurodevelopmental effects more broadly.

From the perspective of the mother–breastmilk–infant “triad” (5), our results provide evidence that milk composition is dynamically changing to support developmental processes in the infant brain. Specifically, the period when inositol peaks in human milk in early lactation matches the roles we identified in promoting synaptic connectivity. Indeed, the remarkably similar profile across global communities of mothers points to important general roles in the newborn diet. *Myo*-inositol in human milk may contribute to the association of breastfeeding with improved brain and cognitive development (4, 32, 33). This would agree with the correlation of higher levels of *myo*-inositol at birth with improved language scores (34). Considering these human data, it will be of interest to determine whether *myo*-inositol’s effects on synapses in the maturing cortex are sustained over time.

Brain inositol declines over time, as measured in the thalamus of term infants at 6.3 ± 1.0 mmol/kg to 3.5 mmol/kg by adulthood (35, 36). With respect to brain functions in adults, lower brain inositol levels were found in patients with major depressive disorder and bipolar disorder (37–40), and genetic alterations in *myo*-inositol transporters are linked to schizophrenia (41). Beneficial effects of *myo*-inositol in depression and anxiety phenotypes have been reported (42, 43). On the contrary, *myo*-inositol has been described to be accumulated in the brain of people with Down’s syndrome and patients diagnosed with Alzheimer’s disease including adults with Down’s syndrome, and may contribute to pathology (44–47). Studies to guide dietary recommendations to balance *myo*-inositol across life stages with consideration of disease conditions are therefore warranted.

Together, our findings highlight breastmilk as a dynamic source of bioactive compounds supporting brain development. These results reveal substantial benefits of *myo*-inositol for the connectivity of the postnatal brain and provide leads for the improvement of pediatric nutrition products.

## Materials and Methods

*SI Appendix* provides an *SI Appendix*, Extended Results and Extended Methods section.

### Global Human Milk Study.

The human milk samples analyzed for inositol content were collected from mothers participating in the GEHM Study at three urban sites in Mexico City, Mexico; Shanghai, China; and Cincinnati, United States. Free *myo*-inositol and total inositol concentration of human milk were determined by HPAEC/PAD. All mothers provided written informed consent, and this study was approved by the Institutional Review Boards of Cincinnati Children’s Hospital Medical Center, the National Institute of Medical Sciences and Nutrition in Mexico City, and Shanghai Children’s Hospital of Fudan University.

### Antibodies.

Primary antibodies and application notes for immunocytochemistry and immunohistochemistry are provided in the *SI Appendix*, Extended Methods.

### Rat Neuronal Culture Preparation and Treatment.

Hippocampi were dissected from rat pups at embryonic day 18 and plated on cover glasses treated with 1 mg/mL poly-l-lysine. The first treatment was at 3 d in vitro (div) with DHA final 4.3 µM or *myo*-inositol final 1 mM or 13 mM.

### Coculture Studies.

Cultures of rat hippocampal neurons were prepared and treated as above and cocultures were prepared by seeding HEK293-expressing Neuroligin 1.

### Human Neuronal Culture Preparation and Treatment.

Human glutamatergic–enriched cortical neurons from induced pluripotent stem cells were purchased from Fujifilm. Treatments started 3 d after plating.

### Immunocytochemistry and Imaging of Cultured Neurons.

Immunostaining of rat hippocampal neurons was performed at 14 div. Confocal images of secondary and tertiary dendrites were analyzed using ImageJ. Immunostaining of human neurons was performed 19 to 24 div after plating. Most synapses formed along the primary dendrite in this system, which was selected for analysis.

### Organotypic Slice Culture Preparation and Treatment.

Organotypic slices of 350 μm thickness were prepared from hippocampi of P3-5 mice. Slices were cultured for 60 d and were then treated with vehicle or *myo*-inositol for a 30-d period by adding it at 1 mM to the medium.

### Immunohistochemistry and Imaging of Organotypic Slice Cultures.

Slices were fixed and processed for immunostaining, followed by confocal imaging of the hippocampal CA3 area. An ImageJ macro was used to create binary masks and quantity puncta and their colocalization.

### Dietary Supplementation of Mice.

All animal procedures undertaken in this study were approved by the Institutional Animal Care and Use Committees and in compliance with NIH guidelines. Studies were performed with C57BL/6J mice from Jackson Labs (Bar Harbor, ME). Beginning on postnatal day 1, pups were fed once daily with 50 mg *myo*-inositol/kg body weight.

### Synapse Staining in the Visual Cortex.

Mice at P35 were transcardially perfused. Fixed brain tissues were sectioned in the coronal plane on a vibrating microtome. Sections containing primary visual cortex were probed with antibodies. Following confocal microscopy, synaptic markers were quantified with the Analyze Particles tool of ImageJ.

### Data Analyses.

Data were acquired and quantified blind to the analyzed condition. Additional information for statistical analyses is provided in the *SI Appendix*, Extended Methods. *P < 0.05, **P < 0.01, ***P < 0.001.

## Supplementary Material

Appendix 01 (PDF)

## Data Availability

Scripts are available on GitHub and links are provided in the *SI Appendix*, Extended Methods. No unique materials were generated for this study.
