# Supplementary material for: The human milk component myo-inositol promotes neuronal connectivity
Source: Proc Natl Acad Sci U S A. 2023 Jul 11;120(30):e2221413120. doi: 10.1073/pnas.2221413120 (PMC10374161; doi:10.1073/pnas.2221413120)
Supplement: Supplementary file 1 — Appendix 01 (PDF) [file pnas.2221413120.sapp.pdf]

PNAS

Supporting Information

The human milk component *myo*-inositol promotes neuronal connectivity

Andrew F. Paquette, Beatrice E. Carbone, Seth Vogel, Erica Israel, Sarah D. Maria, Nikita P. Patil, Saroj Sah, Dhrubajyoti Chowdhury, Ilona Kondratiuk, Beau Labhart, Ardythe L. Morrow, Shay C. Phillips, Chenzhong Kuang, Dirk Hondmann, Neeraj Pandey, and Thomas Biederer

This PDF file includes:

Extended Results ______ page 2

Extended Methods ______ page 3-8

Supplemental Tables S1 to S4 ______ page 9-10

Supplemental Figures S1 to S9 ______ page 11-22

Supplemental References ______ page 23-24

EXTENDED RESULTS

Processes of brain development. Figure 1B in the main text shows stages of early cortical development and maturation. This diagram is based on publications on the profile of synapse density in the human cortex (1, 2) with additional information from non-human primate studies (3), human myelination (4, 5), the radial migration of pyramidal neurons in non-human primates (6, 7), and the tangential migration of interneurons in non-human primates (8). Comprehensive reviews of these processes have been published (9, 10).

***Myo*-inositol supplementation does not alter the density of synaptic specializations in the cortex but enlarges Homer-positive postsynaptic sites.** As described in the Results section of the main paper, quantitative immunohistochemical stainings for synaptic markers were performed in the visual cortex area V1b of mice at P35.

We first analyzed layer II/III, which connects and integrates information across cortical areas. Representative immunostainings for excitatory and inhibitory pre- and post-synaptic markers in layer II/III before and after thresholding are shown in Supplemental Figure 9A. Regarding the density of synaptic puncta in V1b, we measured for VGLUT1, a presynaptic vesicular glutamate transporter that marks mainly intracortical connections (11), that the mean puncta density in control mice was 60 ± 8.5 compared to 58.1 ± 7.9 puncta/100 μm^2 for *myo*-inositol supplemented mice (Supplemental Fig. 9B). Density means for VGLUT1 were not different (N=5 control/4 *myo*-inositol complemented mice, *t*=0.137). As stated in the main Results section, the density of Homer, a post-synaptic marker of excitatory glutamatergic synapses (12), was 38 ± 2.0 for control and 37 ± 2.1 puncta/100 μm^2 for *myo*-inositol, which was not significantly different (N=5/4, *t*=0.1377) (Fig. 3C).

We also analyzed the density of inhibitory synaptic sites in layer II/III of V1b (Supplemental Fig. 9A, B). The inhibitory postsynaptic scaffolding protein Gephyrin showed average densities of 19.8 ± 1.92 for control and 20.4 ± 1.62 puncta/100 μm^2 for *myo*-inositol treated mice, which were not different (N=6/5, *t*=0.243). For the inhibitory presynaptic marker Vesicular GABA Transporter (VGAT), control puncta had an average density of 7.6 ± 1.15 while *myo*-inositol puncta density was 7.3 ± 0.72 puncta/100 μm^2, with no difference between treatments (N=6/5, *t*=0.161).

In the same set of images from layer II/III of V1b, we quantified the size of synaptic puncta. VGLUT1 puncta were not significantly different, with control mice having an average size of 0.085 ± 0.009 μm^2 compared to 0.097 ± 0.010 μm^2 for *myo*-inositol complemented mice (N=5/4, *t*=0.897, not significant) (Supplemental Fig. 9C). As stated in the main Results section, Homer puncta size was increased from 0.129 ± 0.015 μm^2 in control animals to 0.192 ± 0.017 μm^2 for *myo*-inositol complemented mice (Fig. 3D) (two-tailed unpaired *t*-test; N=5/4, *t*=2.764, p=0.028). This increase occurred across all size populations as supported by analysis of Homer puncta size distribution (Kolmogorov–Smirnov test, N=5 control/4 *myo*-inositol complemented mice, *t*=0.154, p=0.032) (Supplemental Fig. 9D). For the inhibitory postsynaptic marker Gephyrin, we found an average puncta size of 0.100 ± 0.003 μm^2 for control versus 0.096 ± 0.004 μm^2 for *myo*-inositol complemented mice (N=6/5, *t*=0.748, not significant) (Supplemental Fig. 9C). VGAT average control puncta size was 0.080 ± 0.006, while average puncta size in *myo*-inositol complemented mice was 0.088 ± 0.005 μm^2 (N=6/5, t=1.087, not significant).

We also quantified VGLUT2, a marker of excitatory thalamocortical projections (13), in the primary thalamocortical input layer IV (Supplemental Fig. 9E). VGLUT2 puncta showed a mean density of 15.9 ± 0.45 puncta/100 μm^2 in control mice, and *myo*-inositol treated animals had an undistinguishable mean density of 16.1 ± 0.51 puncta/100 μm^2 (N=6/5, *t*=0.173) (Supplemental Fig. 9F). VGLUT2 puncta size in layer IV was unaltered upon *myo*-inositol complementation (N=6/5, t=0.656) (Supplemental Fig. 9G).

Additionally, we measured the densities and size of synaptic markers in layer V of visual cortex V1 (Supplemental Fig. 10). The density of the synaptic markers VGLUT1, Gephyrin, and VGAT (Supplemental Fig. 10A) and their size (Supplemental Fig. 10B) analyzed in V1b layer V were not significantly changed upon *myo*-inositol complementation. As stated in the main Results section, Homer puncta were enlarged in layer V, from 0.134 ± 0.011 for control to 0.200 ± 0.007 μm^2 for *myo*-inositol complemented animals (N=5/4, *t*=4.630, p=0.002) (Fig. 3D), with no effect on Homer puncta density (Fig. 3C).

EXTENDED METHODS

Global human milk study. The Global Exploration of Human Milk (GEHM) multi-site study to detail variation in the composition of human milk geographically and over the course of lactation was described previously (14-16). All mothers provided written informed consent, and this study was approved by the Institutional Review Boards of Cincinnati Children's Hospital Medical Center, the National Institute of Medical Sciences and Nutrition in Mexico City, and Shanghai Children's Hospital of Fudan University.

Mothers of term, singleton infants ≥2500 g birth weight and planning to breastfeed ≥75% for at least 3 months were recruited at three urban sites in Mexico City, Mexico; Shanghai, P.R. of China; and Cincinnati, United States. Milk samples were collected at 2, 4, 13, 26, and 52 weeks, as long as mothers continued breastfeeding. Samples were collected during morning hours by emptying an entire breast via an electric pump, and samples were refrigerated and prepared for cryogenic storage no later than 4 hours after pumping (17). For inositol analysis, 30 mothers (N=10 per site) with milk collected across all 5 timepoints from 2 to 52 weeks of lactation were chosen for study inclusion, with a total of 150 milk samples analyzed.

Free *myo*-inositol and total inositol concentration of human milk were determined by HPAEC/PAD as described (18), with modifications. For adaptation to small volume milk samples, 0.5 ml of human milk was diluted to 10 ml with water and sonicated 30 min before free *myo*-inositol analysis. For analysis of total inositol including bound forms like phosphatidylinositol, 1.0 ml milk was combined with 4 ml of water and 5 ml of HCl, autoclaved at 121 °C for 6 h, neutralized with 6 ml 10N NaOH, and brought to 50 ml with water. Method suitability for quantitation of free and total myo-inositol in human milk with these modifications was demonstrated by accuracy/precision studies before GEHM milk analyses. Daily infant inositol intakes from human milk were modeled per lactation stage using a mean of total 580, 630, 760, 780, and 780 ml of human milk fed per day at 2, 4, 13, 26, and 52 weeks, respectively, as adapted from stable isotope human milk intake studies (19) and converted to volume using a reference human milk density of 1.03 g/l.

Statistical analysis of inositol levels in mothers from the United States, Mexico, and China over one year of lactation was conducted using R (version 3.6.1) with the following additional libraries: lattice (0.20-38), rstatix (0.4.0), and tidyr (1.0.0). Normality was observed for all country-week combinations except for Chinese mothers at week 13 ($p = 0.002$) and US mothers at week 26 ($p = 0.04$) based on the Shapiro-Wilk test, and data transformation was deemed unnecessary. Data satisfied the assumptions for homogeneity of variances (Levene's test), equality of covariances (Box's M Test), and sphericity (Mauchly's test). Inositol data was modeled in a factorial repeated measures analysis of variance (rmANOVA) treating country as a categorical factor between mothers, week of lactation as a repeated continuous factor within mothers, and the country by week interaction, with p-value <0.05 taken to be significant.

Antibodies. Primary antibodies and application notes for immunocytochemistry (ICC) and immunohistochemistry (IHC) are provided in the table below. Secondary antibodies, including isotype-specific ones, were conjugated to Alexa dyes 405, 488, 555, and 647 (Thermo Fisher).

Target	Vendor	Cat. No.	RRID	Host and clonality	ICC	IHC cortex	IHC slice cultures
Bassoon	Enzo	VAM-PS003	AB_2313991	Mouse monoclonal IgG2a	1:500 (o/N at 4 °C)		1:500 (72 h at 4 °C)
VGLUT1	Millipore	AB5905	AB_2301751	Guinea pig polyclonal	1:500 (o/N at 4 °C)	1:1000 (48 h at 4 °C)	
VGLUT2	Millipore	AB2251	AB_1587626	Guinea pig polyclonal		1:1000 (48 h at 4 °C)	
Homer	Synaptic Systems	160 003	AB_887730	Rabbit polyclonal	1:600 (o/N at 4 °C)	1:500 (48 h at 4 °C)	

PSD-95	Cell Signaling	3450	AB_2292883	Rabbit monoclonal			1:500 (72 h at 4 °C)
PSD-95 FluoTag-X2	Synaptic Systems	N3702-AF647	AB_2936216	Camelid nanobody AlexaFluor 647 conjugated	1:500 (o/N at 4 °C)		
VGAT	Synaptic Systems	131 003	AB_887869	Rabbit polyclonal	1:500 (o/N at 4 °C)	1:5000 (36 h at 4 °C)	
Gephyrin	Synaptic Systems	147 111	AB_887719	Mouse monoclonal IgG1		1:400 (72 h at 4 °C)	
MAP2	Millipore	MAB3418	AB_94856	Mouse monoclonal	1:1000 (o/N at 4 °C)		
MAP2	Millipore	AB5543	AB_571049	Chicken polyclonal	1:1000 (o/N at 4 °C)		1:1000 (o/N at 4 °C)
Iba1	Wako	019-19741	AB_839504	Rabbit polyclonal			1:1000 (72 h at 4 °C)

Human neuronal culture preparation and treatment. Human glutamatergic-enriched iCell GlutaNeurons derived from induced pluripotent stem (iPS) cells were purchased fully differentiated from Fujifilm (#ICELL GNC 01279; Santa Ana, CA) and cultured per manufacturer's instructions. These are fully differentiated and post-mitotic and highly pure glutamatergic neurons (>90%) on day 3 post-thaw as determined by the percentage of Tuj+/Nes- cells through flow cytometry per manufacturer's analysis. The iPSC line from which they are differentiated by the manufacturer is registered at https://hpscreg.eu/cell-line/CDIi001-A

For treatments, DHA was applied at 20 mM and *myo*-inositol at 2.0 mM starting 3 days after plating and compounds were added every 2 days upon medium replacement. Human neurons were cultured in BrainPhys Neuronal Medium (STEMCELLTechnologies, #05790) that includes 0.07 mM inositol and lacks DHA (20), in presence of iCell Neural Supplement B and iCell Nervous System Supplement (FUJIFILM Cellular Dynamics, #R1149), which lack *myo*-inositol and DHA. Neurons were fixed 19-24 div after plating.

Rat neuronal culture preparation and treatment. Neuronal cultures were prepared from Sprague-Dawley rat embryos obtained from Charles River Laboratories (Willimantic, CT). Hippocampi were dissected from pups at embryonic day 18. Neurons were dissociated in 0.05 % trypsin and washed 3 times in adhesion media (DMEM, Invitrogen 11965-118; 10 % horse serum, Invitrogen 26050-088; 0.2% Penicillin/Streptomycin Invitrogen 15140-122 by volume) before plating at a density of 60,000 cells on 12 mm, no. 1.0, thickness 90-120 µm cover glasses (Carolina Biological Supply Company, Assistent 633009) treated with 1 mg/ml poly-l-lysine (Sigma, P1274). Plated neurons were incubated at 37 °C in 1 ml adhesion media for 4-6 h and the media was then exchanged for 1 ml NB medium (Neurobasal, Invitrogen 21103-049; 3 % B27 supplement, Invitrogen 17504-001; 0.5 % Penicillin/Streptomycin, Invitrogen 15140-122; 1 % Glutamax supplement, Invitrogen 35050-061). After 2 days *in vitro* (div), mitosis was inhibited using Cytosine β-D-arabinofuranoside (Ara-C) at 2 µM to prevent glial growth. The medium containing Ara-C was aspirated at 3 div and replaced with 0.5 ml NB medium. Neurobasal medium includes 0.04 mM inositol and lacks DHA, see https://www.thermofisher.com/us/en/home/technical-resources/media-formulation.251.html The culture medium has an osmolarity of 279 - 309 mOs and *myo*-inositol addition would only marginally change the osmolarity of the culture medium.

For DHA treatment, a stock of 76 µM DHA (Sigma D2534) was prepared in DMSO under N_2 in a sealed chamber to prevent oxidation (21), and stored in single-dose aliquots at -80 °C until use. Immediately prior to neuron addition, the DHA stock was diluted to 8.75 µM in Neurobasal (NB) medium containing 0.06 %

Vitamin E (a-tocopherol, Fisher Scientific AC428120250), and added to neurons at final 4.3 μM. All treatments were performed in presence of 0.06 % Vitamin E as antioxidant for the positive control DHA. *Myo*-inositol (Sigma I5125; PubChem SID 57654297) was prepared immediately prior to addition to neurons at 26 mM and 2 mM in NB medium containing 0.06 % Vitamin E, sterile-filtered, and added to neurons at final concentrations of 13 mM and 1 mM. Standard NB culture medium contains 40 μM *myo*-inositol. For control treatments, NB medium containing 0.4 % DMSO (Thermo Fisher D12345) and 0.06 % Vitamin E was added to cultures to a final concentration of 0.2 % DMSO.

The first treatment of cultured neurons was at 3 div, when Ara-C was aspirated and replaced with fresh NB medium or NB medium containing DMSO, DHA, or *myo*-inositol at the final concentrations stated above. On 7 div and 10 div, one-half of the medium was removed and replaced with fresh NB medium containing DMSO, DHA, or *myo*-inositol as described above. Neurons were fixed on 14 div.

Co-culture studies. Cultures of rat hippocampal neurons were prepared and treated on 3 and 7 div as described above and co-cultures were prepared as described (22). Briefly, when neurons reached 7 div, 70% confluent HEK293 cells were transfected using polyethylenimine (23) with pcDNA3.1 ECFP alone as a transfection marker control, or pcDNA3.1 ECFP and pCMV-NL14 expressing Neuroligin 1 (24) (a gift from Dr. T. Südhof), at approximately 0.4 picomol plasmid per 9.5 cm^2 well. Transfected HEK293 cells were seeded one day later onto 8 div neurons at a density of 5,000 HEK cells per 12 mm coverglass. Ara-C was added at 2 μM upon seeding the HEK cells onto the neural cultures to prevent overgrowth. After 48 h, co-cultures were fixed on 10 div and processed for immunostaining.

Immunocytochemistry and imaging of cultured human and rat neurons. Immunostaining of cultured rat neurons was performed at 14 div, except for the analysis of Homer and PSD-95 co-localization described below. Following fixation for 15 min at RT with 4% PFA/4% sucrose in PBS, neurons were washed with PBS, blocked, and stained with primary antibodies diluted in 3% fetal bovine serum (FBS)/0.01% Triton-X 100 in PBS, and incubated overnight at 4°C. Primary antibody concentrations are provided in the table. Secondary antibodies were applied at 4° C for 4 h. Coverslips were washed in PBS and mounted with Aqua/PolyMount (Fisher, NC9439247). Confocal microscopy was performed on a Leica TCS SP8. Images were acquired with an ACS APO 63x oil lens with 1.3 NA, using the same settings for each condition. Secondary and tertiary dendrite segments between 20-50 μm length were selected for analysis.

For immunostaining analysis of Homer co-localization with PSD-95 and Bassoon, mature rat hippocampal neurons were fixed using cold methanol at -20° C for 10 min. Neurons were used at 19 div, when postsynaptic sites are mature. Following fixation, neurons were washed with PBS, blocked with 5% BSA/4% normal goat serum in PBS for 1 h at RT, and primary and secondary antibody incubations were performed in 2% BSA in PBS. Secondary antibodies were applied at 1:2000 for 1 h at RT. Coverslips were washed in PBS and mounted with Aqua/PolyMount. Confocal images were acquired on Zeiss LSM800 confocal microscope with a 63x objective plus 1.5x digital zoom and maximum intensity projection images were used for analysis as described above.

Immunostaining of human neurons was performed at 19-24 div following the procedure described above for cultured rat neurons at 14 div. The majority of synapses at the time point of 19-24 div after plating analyzed here were formed along the primary dendrite, and we selected these segments for analysis.

Quantification of images from cultured neurons was performed with the researcher blind to the condition during image acquisition and analysis. Images were analyzed with ImageJ using a custom-written macro (Dr. Lai Ding, NeuroTechnology Studio and Program for Interdisciplinary Neuroscience, Brigham and Women's Hospital) as in past studies (21, 25). Briefly, images were converted to maximum projections, ROIs were manually selected by tracing MAP2-positive dendrites, length was measured, and thresholds determined per channel based on the control condition. The macro then generated for each channel a mask based on the threshold to define puncta and saved puncta counts, and sizes and counted puncta of each channel that colocalize over at least 25% of their area with the other. The script is available on GitHub https://doi.org/10.5281/zenodo.7883923 (26).

Organotypic slice cultures preparation and treatment. Organotypic slices of 350 μm thickness were prepared as described from hippocampi of P3-5 mice (27, 28), and cultured at 33° C. Medium was changed every 3-4 days. For synaptic marker analysis, slices were cultured for 60 days and then treated with *myo*-inositol for 30 days by adding it at 1 mM to the medium every time it was changed. For microglia analysis and Iba1 staining, slices were cultured for 16 weeks and then treated with *myo*-inositol for 14 days by adding it at 1 mM to the medium every time it was changed. Slice cultures were processed for immunostaining after this period. Control slices were treated with medium to which vehicle was added.

Immunohistochemistry and acquisition and analysis of confocal images from organotypic slice cultures. Organotypic slices were fixed in 8% PFA in PBS, then stored in PBS at 4 °C, protected from light. Slices were permeabilized in 1% Triton X-100 in PBS for 18 hours at 4 °C, then blocked in 1 ml blocking buffer (20% BSA in PBS) for 4 hours at room temperature. All solutions were filtered. The Teflon inserts (Millipore) containing the slice cultures were cut out and placed on top of 1 ml PBS on coverslips on a 6-well plate lid. The integrity of the PBS droplet was maintained by surrounding the coverslip using a PAP pen. Primary antibodies were diluted in dilution buffer (5% BSA in PBS) as described in the antibody table. The Homer antibody from Synaptic Systems (#160 003) did not yield high-quality staining in organotypic slice cultures and we used the PSD-95 antibody from Cell Signaling (#3450) in this preparation. All slices were incubated in 350 µl of diluted primary antibody per coverslip for 72 hours at 4 °C on a rocker at slow speed and shielded from light. This was followed by four washes in PBS. Secondary antibodies (1:500 in 350 µl antibody dilution buffer) were applied for 72 hours at 4 °C on a rocker at slow speed and shielded from light. Samples were then washed four times with 1000x DAPI diluted in the third PBS wash. Wash steps were carried out at room temperature on a rocker and shielded from light. Teflon sheets containing the slice cultures were then transferred into glass microscope slides (Fisher), dried, and cover-slipped (Fisher) with Aqua-Mount (Thermo-Scientific). Z-stack projections of 4-5 μm thickness were acquired in the hippocampal CA3 stratum lucidum layer with a Leica SP8 confocal microscope with an HC PL APO CS2 63x/ 1.40 NA oil objective (Leica). Layer identification was based on labeling the CA3 stratum pyramidale with DAPI. Laser power, gain, and offset were chosen for each round of IHC based on the average of manually selected values of several of the control-treated slice cultures and were kept constant within a given imaging session. Each 12-bit 1024x1024 image was acquired at a scanning speed of 400 Hz.

For quantification of synaptic puncta obtained from slices cultured for 90 days and the extent of their co-localization, 400x400 pixel ROIs were cropped from each image to analyze areas with synaptic puncta and lacking cell bodies. A custom-written ImageJ macro was used to apply a threshold for background subtraction. This threshold was chosen using twice the average size of the largest non-puncta object in the control images. This threshold was used as an input radius for the rolling ball size in which the rolling ball subtracted the radius value in each spot over the image. The macro created binary masks of each channel which were then manually overlayed on top of the original image of the complementary channel to quantify the total number of puncta in each channel as well as the percent colocalization of the synaptic puncta using the area and area fraction analysis in the built-in ROI manager in ImageJ. The script is available on GitHub https://doi.org/10.5281/zenodo.7865411 (29).

For quantification of microglia density and Iba1 signal intensity in organotypic slices cultured for 18 weeks, a rolling ball background subtraction was performed in ImageJ using a 240 pixel setting. Rolling ball background subtraction was based on the average smallest size of apparent Iba1 positive cell fragments in the control sum slice projection images, which was 22 μm^2. Built-in Otsu thresholding in ImageJ was then applied to each image, with automated thresholding performed for each image to account for variability in Iba1staining. The built-in Analyze Particles function in ImageJ was used to record the ROIs of each detected Iba1 positive cell and create outlines/masks of these ROIs. ROIs were created using the Analyze Particles function based on a minimal cell area of 50 μm^2. This cut-off excluded incompletely imaged cells including those that appeared partially out of the acquired optical sections. The average apparent area of the Iba1 positive cells that remained for control and *myo*-inositol treatment images after the cut off applied was 2400 and 1500 μm^2, respectively. Within each ROI, the number of Iba1 positive cells above the minimal area threshold was counted, and the average integrated intensity of Iba1 signal of these cell areas was measured.

Dietary supplementation of mice. All animal procedures in this study were approved by the Institutional Animal Care and Use Committees and in compliance with NIH guidelines. *In vivo* studies were performed with wild type (WT) C57BL/6J mice from Jackson Labs (Bar Harbor, ME). Larger litters were culled to have no more than 8 mice. Female breeders were retired after giving birth to and nursing their fifth litter.

Myo-inositol (Sigma I5125; PubChem SID 57654297) was dissolved weekly in sterile, deionized H_2O to a final concentration of 50 mg/ml and this working solution was stored at room temperature. Beginning on postnatal day 1 (P1), mouse pups were fed once daily. Feeding was performed on most days in the morning. Animals received *myo*-inositol at 50 mg/kg body weight. Littermate controls were fed equal volumes of sterile, deionized H_2O. Feeding involved pipetting the solution into the back of the mouth with a gel loading pipette tip to trigger swallowing. Post-weaning, the mice were separated by sex and fed regular chow (Envigo, Teklad Global 18% protein, 2918) *ad libitum* in addition to continued *myo*-inositol supplementation or vehicle control delivery.

Myo-inositol amounts for dietary supplementation were determined as follows. Average weight and milk consumption for a 2-week-old human infant are 3.4 kg and 800 ml/day. The concentration of *myo*-inositol in human milk at this stage is 180 mg/l (Fig. 1), corresponding to a *myo*-inositol intake of approximately 45 mg/kg bodyweight/day of infants receiving human milk. The added amount of dietary supplemented *myo*-inositol in mouse pups is therefore comparable to the total intake of a newborn. The amount of inositol provided to mouse pups by their mothers was calculated as follows. Mouse milk consumption at P7 is 0.88 g/day (30), average body weight is 3.9 g, and *myo*-inositol concentration in mouse milk is 800 mg/g, similar to rat (31). This equates to a *myo*-inositol intake of mouse pups of 180 mg/kg bodyweight/day via milk. Supplementation at 50 mg *myo*-inositol/kg body weight corresponded to an increase in *myo*-inositol intake by approximately 30% over the amount of 180 mg/kg bodyweight/day mouse pups take up via milk.

Immunohistochemistry and acquisition and analysis of confocal images from mouse brain tissue. Mice were transcardially perfused using a 27G needle with cold filtered 1x Phosphate Buffered Saline (PBS), pH 7.3, until the liver cleared. Perfusion was done with a peristaltic perfusion pump (GE Healthcare), using approximately 25 ml, and being followed by 10 ml of 4% paraformaldehyde (PFA) in PBS, pH 7.3. Brain tissues were then removed and drop fixed for 24 h at 4 °C in 4 % PFA in 1x filtered PBS before being stored in PBS at 4 °C, all shielded from light. Fixed brain tissues were sectioned in the coronal plane at 75 µm thickness on a vibrating microtome (Vibratome 1500, Harvard Apparatus, Holliston, MA). Sections containing primary visual cortex were visually identified by locating the posterior boundaries of the CA3 and referencing the Allen Mouse Brain Atlas https://mouse.brain-map.org/static/atlas

For quantification of individual synaptic puncta detected as above, sections containing the visual cortex were obtained at P35 as described above. Antigen retrieval was carried out by placing individual sections in 1 ml of 73 °C citrate buffer pH 6.2 for 35 minutes, cooling to RT, then washing three times in PBS. Sections were blocked in 1 ml blocking buffer (3 % normal horse serum, 0.1% Triton X-100 in PBS) for 1 h. Primary antibodies were diluted in antibody dilution buffer (3 % normal horse serum, 0.05 % Triton X-100 in PBS). Up to three sections were incubated in 250 µl of diluted primary antibody per well of a 24-well plate for 36-72 h at 4° C. Primary antibody concentrations and probing times are provided in the section Antibodies. This was followed by three washes in PBS. Secondary antibody (1:500 in 250 µl antibody dilution buffer) was applied for 2-3 h at RT (VGLUT2, VGAT, Gephyrin) or 12 hours at 4 °C (VGLUT1, Homer). After secondary antibody incubation, the sections were washed three times as before. All incubation and wash steps were carried out at RT on an orbital shaker and shielded from light unless otherwise noted. Sections were then floated onto glass microscope slides (Fisher), dried, and cover-slipped (Fisher) with CFM3 (Citifluor, Hatfield, PA). Tile scans of the entire binocular V1 (V1b) from a single hemisphere were imaged with a Leica SP8 confocal microscope with an HC PL APO CS2 63x/1.40 Oil objective (Leica) and a HyD detector. Single optical sections of 1 µm were obtained. Laser power, gain, and offset were chosen for each round of IHC based on the average of manually selected values of several control sections and kept constant for a given experiment. 12-bit 2048x2048 images were acquired at a scanning speed of 100 Hz. For quantification, 10% was cropped off all sides of the images to correct for tile scan overlap and avoid quantifying the same region twice. Boundaries between cortical layers were drawn based on the method of Tomassy et al. (32). Briefly, a rectangle spanning from the pia to the white matter

tracts orientated perpendicular to the white matter was selected. Layer boundaries were drawn within this rectangle and extrapolated across the merged image running parallel to the curvature of the brain surface. Tiles with at least 50% of their area contained within layers II/III, IV, or V were included in the analysis. For tiles that spanned multiple layers, the layer boundaries were added as an image overlay. Single layer ROIs were outlined for each of the selected images using the layer boundaries as a guide where needed. Layers II and III were treated as a single layer. Within square ROIs, Robust Automatic Thresholding Segmentation (RATS) was done for each channel. Noise threshold was determined using the standard deviation of background intensities from two manually outlined somas per image, where signal intensity was minimal. Puncta size and number were quantified with the built-in Analyze Particles tool of ImageJ with no size-based exclusions. A custom Python script was used to average puncta from different images within a section and across multiple sections for each animal. Cumulative distribution frequency plots and statistic distribution plots for the permutation tests were also created in Python.

For quantification of the extent of Homer co-localization with PSD-95, sections containing the visual cortex were obtained at P50 as described above. The above staining protocol was used with minor modifications. Fixed brain tissue was sectioned at 50 μm thickness on a Vibratome 1500 (Harvard Apparatus). Blocking buffer was 5% normal horse serum, 0.1% Triton X-100 in PBS) and antibody dilution buffer was 5% normal horse serum, 0.01%Triton X-100 in PBS. Sections were incubated for 24 h at RT. Secondary antibodies were applied for 24 hours at 4° C. After secondary antibody incubation, the sections were washed three times as before. Sections were cover-slipped with ProLong Diamond Antifade Mountant. Images from 5 optical sections containing primary visual cortex V1 at an interval of 0.25 μm were acquired on a Zeiss LSM800 confocal microscope with a 63x objective at 1.0 μm optical thickness. Optical maximum-intensity projection images were generated. Puncta colocalization analysis was performed in ImageJ. For each acquired image, two ROIs of 10x10 μm^2 were chosen, avoiding regions with cell bodies. Homer and PSD-95 channels for all ROIs were converted to grayscale and the same threshold was set for each channel and was used to generate corresponding masks. Colocalization was assessed by overlaying the mask of traced puncta of one channel onto the unprocessed other channel and overlapping puncta were scored as colocalized with the aid of the cell counter plugin.

Data analyses. Data acquisition and quantifications were performed blind to the analyzed condition. Statistical analyses were performed with GraphPad Prism software version 8.2.1. Data are presented as mean, with errors corresponding to the standard error of the mean, except where noted. Violin plots show frequency distribution curves of all data showing the median and quartiles, using the Kernel density method that results in a distribution that extends above the largest value and extends below the smallest value. For comparison of means between two groups, unpaired two-tailed *t*-tests were used, and standard errors are noted unless stated otherwise. One-way ANOVA was used to compare multiple groups simultaneously. Cumulative distribution frequency plots were analyzed with an exact permutation test. Briefly, the two-sample Kolmogorov-Smirnov test statistic was calculated with the actual treatment assignments of the animals and then for every permutation of treatment assignment with the same group sizes. The p-value is equivalent to the fraction of all generated test statistics greater than or equal to the observed test statistic. * $p<0.05$, ** $p<0.01$, *** $p<0.001$

For cumulative distribution function (CDF) analyses of synaptic puncta sizes measured after immunohistochemistry in the cortex, we employed a two-sample Kolmogorov-Smirnov test, a non-parametric test that reports the greatest vertical distance between two CDF plots. To test for significance, we used a block-exchangeable exact permutation test (33) with the two-sample Kolmogorov-Smirnov test statistic as the dependent variable. This approach tests the null hypothesis that the treatment assignment has no predictive value and therefore randomly reassigning data points to one treatment or the other will not affect the results. The total number of animals per experiment was 5 control/4 *myo*-inositol complemented (VGLUT1 / Homer) or 6/5 (VGLUT2, VGAT, Gephyrin). This allowed us to perform an exact test, i.e. we tested all possible permutations of data points. Because many observations came from each animal, we permuted at the animal level, the same as original treatment allocation, thereby preserving the dependent structure of the data. The only assumption of a permutation test is exchangeability, which is satisfied by keeping observations from the same animal in the same permutation block.

SUPPLEMENTAL TABLES

		Cincinnati, USA	Shanghai, China	Mexico City, Mexico
	n	*10*	*10*	*10*
Maternal	Age at delivery, *years*	32.4 ± 4.3	28.9 ± 4.4	25.9 ± 5.0
	Pre-pregnancy BMI	27.9 ± 5.4	19.9 ± 2.5	24.1 ± 4.6
	Gestational weight gain, *kg*	10.1 ± 3.0	16.5 ± 4.9	10.0 ± 4.3
	Parity	1.8 ± 2.3	0.1 ± 0.3	1.3 ± 1.1
	Delivery method, *n (%) vaginal*	8 (80%)	3 (30%)	6 (67%)*
Infant	Gender, *n (%) male*	4 (40%)	6 (60%)	6 (60%)
	Gestational age, *weeks*	40.1 ± 0.6	40.0 ± 0.8	38.8 ± 0.6
	Birth weight, *kg*	3.8 ± 0.4	3.4 ± 0.4	3.1 ± 0.2

Supplemental Table 1. Demography of GEHM mother-infant dyads (n=30) with human milk characterized for inositol concentration. Values represent mean ± SD unless indicated. * n=9 for the delivery method for Mexico City mothers.

	Week 2	Week 4	Week 13	Week 26	Week 52	ANOVA p-value
**Free *myo*-inositol** mean ± SD (mg/l)	180.1 ± 40.9	171.6 ± 37.9	135.4 ± 27.3	112.0 ± 27.8	79.7 ± 22.6	week of lactation <0.01<br>country = 0.35<br>country x WOL = 0.52
**Free *myo*-inositol intake** mean (mg/day)	104.5 ± 23.7	108.1 ± 23.8	98.8 ± 19.9	87.4 ± 21.7	62.2 ± 17.6	
**Total inositol** mean ± SD (mg/l)	192.4 ± 42.7	182.9 ± 39.5	144.4 ± 28.8	120.1 ± 27.6	87.6 ± 23.5	week of lactation < 0.01<br>country = 0.36<br>country x WOL = 0.55
**Total inositol intake** mean (mg/day)	111.6 ± 24.8	115.2 ± 24.9	105.4 ± 21.0	93.7 ± 21.5	68.3 ± 18.4	

Supplemental Table 2. Free *myo*-inositol and total inositol concentration and modeled infant intake from human milk over weeks of lactation. Values were obtained from milk collected by ten mothers per site in Shanghai, China; Mexico City, Mexico; and Cincinnati, United States, over lactation. Total inositol reflects free *myo*-inositol and bound inositol forms including inositol phosphate and phosphatidylinositol. Infant intake of inositol from human milk was estimated assuming lactation stage-specific milk intake (19). Because the volume of human milk taken by the infant increases as the infant develops (580 to 800 ml from 2 to 52 weeks), modeled intakes of both free and total inositol are relatively stable for the infant from 2 through 13 weeks of age and modestly decrease at 3 months of life before decreasing markedly at 12 months. WOL, week of lactation. P-values ≤ 0.05 were considered significant group effects.

Factor	p-value	Effect size
Country	0.35	USA: 1.6%; China: 8.3%; Mexico: -9.9%
Week of Lactation	<0.01	-55.8%
Country x Week of Lactation	0.52	USA: 9.2%; China: 9.7%; Mexico: -19.4%

Supplemental Table 3. Analysis of variance for free *myo*-inositol in GEHM human milk. For country, the effect size was calculated as percent difference between country mean and global mean at baseline (2 weeks). Effect size for week of lactation was percent difference between mean global inositol at 2 and 52 weeks. For country-by-week interaction, the effect size is reported as the percent difference between country slope and overall global slope over time.

Factor	p-value	Effect size
Country	0.36	USA: 2.8%; China: 7.1%; Mexico: -9.9%
Week of lactation	<0.01	-54.47%
Country x Week of lactation	0.55	USA: 10.3%; China: 7.8%; Mexico: -18.6%

Supplemental Table 4. Analysis of variance for total inositol in GEHM human milk. For country, effect size was calculated as percent difference between country mean and global mean at baseline (2 weeks). Effect size for week of lactation was percent difference between mean global inositol at 2 and 52 weeks. For country-by-week interaction, the effect size is reported as percent difference between country slope and overall global slope over time. Concentrations of free vs. bound fractions of inositol within an individual milk sample showed only a very weak positive correlation (R2 = 0.05).

SUPPLEMENTAL FIGURES

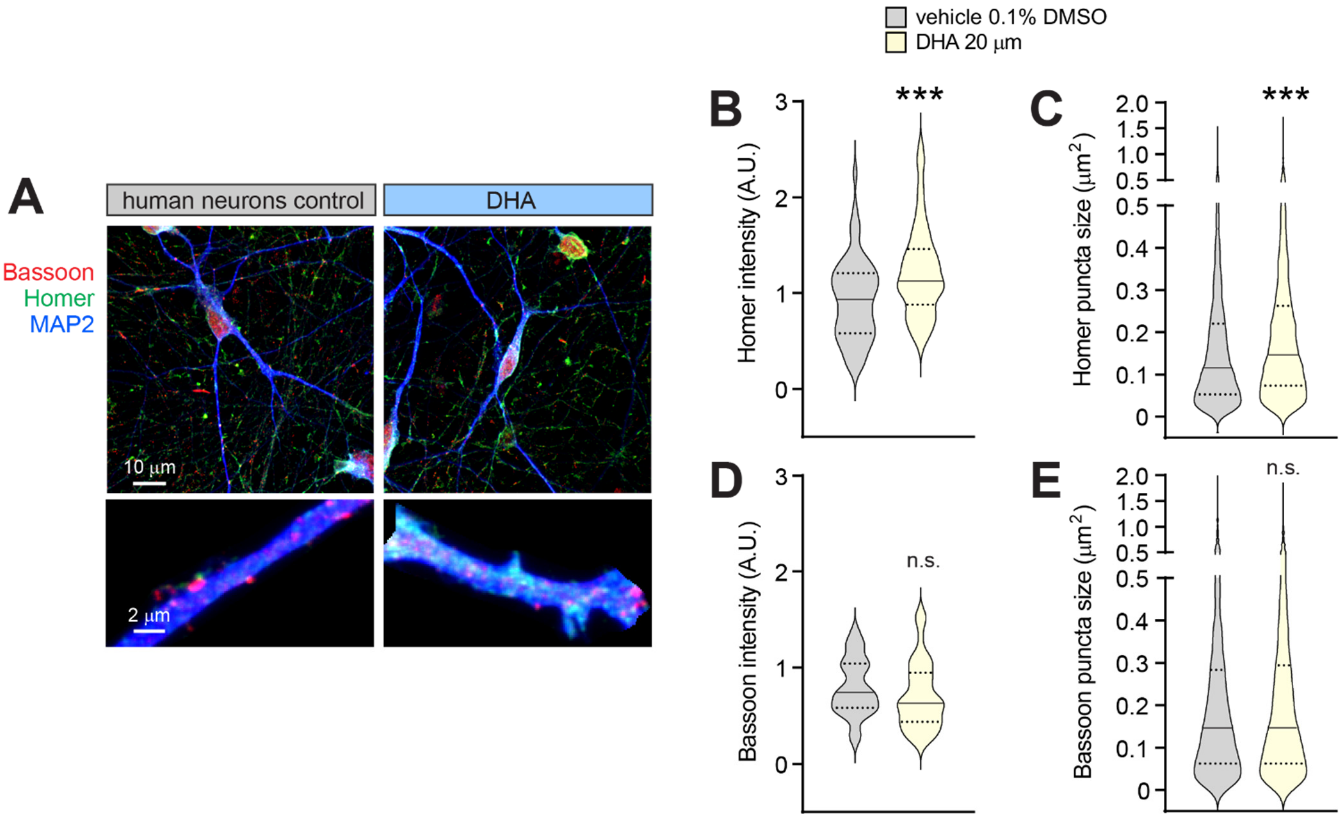

Supplemental Figure 1. DHA promotes postsynaptic maturation of human glutamatergic neurons

(A) Top, representative confocal images of human glutamatergic-enriched cortical neurons at 24 div after plating (left), or after treatment with DHA at 20 μM (right). Immunostainings were performed for presynaptic Bassoon (red), excitatory postsynaptic Homer (green), and dendritic MAP2 (blue). Bottom, panels show enlarged dendritic segments.

(B, C) Quantification of images as in (A) determined that DHA increases in human glutamatergic neurons the abundance of postsynaptic Homer measured as immunostaining intensity per dendritic area (B) and the size of Homer-positive specializations (C). Violin plots show data distribution. Solid lines mark the median and dotted lines the quartiles. Asterisks show statistical differences of mean values, which are not plotted. (Student's t-test, two-tailed unpaired; dendritic segments from N=86 control/71 *myo*-inositol treated neurons) *** p<0.001

(D, E) DHA treatment did not significantly increase presynaptic Bassoon abundance measured as immunostaining intensity (D) and size of Bassoon-positive sites (E). n.s., not significant.

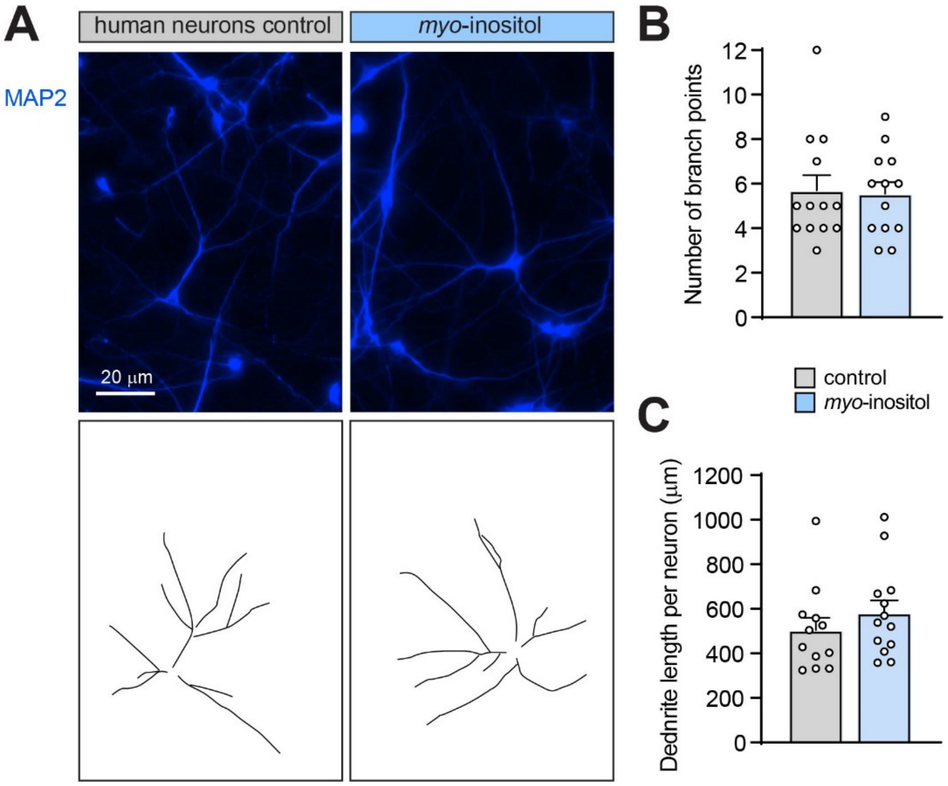

Supplemental Figure 2. *Myo*-inositol treatment does not alter the dendritic differentiation of human glutamatergic neurons

(A) Top, confocal overview images of human glutamatergic-enriched cortical neurons at 24 div after plating (left), or after treatment with myo-inositol at 2 mM (right). Immunostainings were performed for the dendritic MAP2 (blue). Bottom, panels show traced MAP2-positive dendritic segments.

(B) Quantification of images as in (A) showed that *myo*-inositol supplementation did not alter the number of dendritic branch points. (Student's *t*-test, two-tailed unpaired; *n*=13 neurons per condition)

(C) Quantification of images as in (A) showed that *myo*-inositol supplementation did not alter the length of dendrites. (Student's *t*-test, two-tailed unpaired)

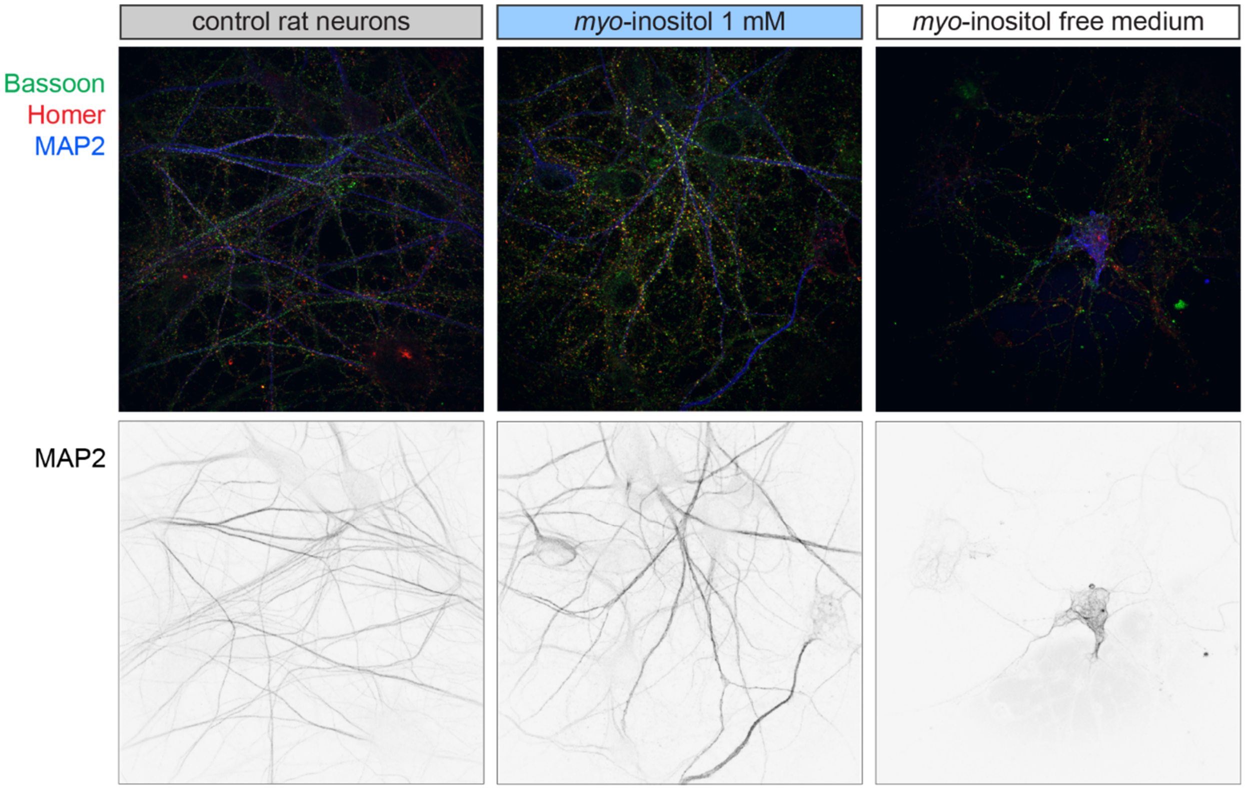

Supplemental Figure 3. *Myo*-inositol is required for neuronal health

Representative confocal images show cultures of dissociated rat hippocampal neurons at 14 div cultured in standard Neurobasal medium containing 0.04 mM inositol as control (left), medium supplemented with *myo*-inositol at 1 mM (center), or custom-made medium lacking inositol (right). Immunostaining for presynaptic Bassoon (green), postsynaptic Homer 1 (red), and dendritic MAP2 (blue) was performed. Neurons grown without inositol appeared incompletely differentiated with blebbed neurites, and had fewer synapses. Differentiation impairments in absence of inositol began to appear at 3-4 div. Top, overview images. Bottom, MAP2 signal alone.

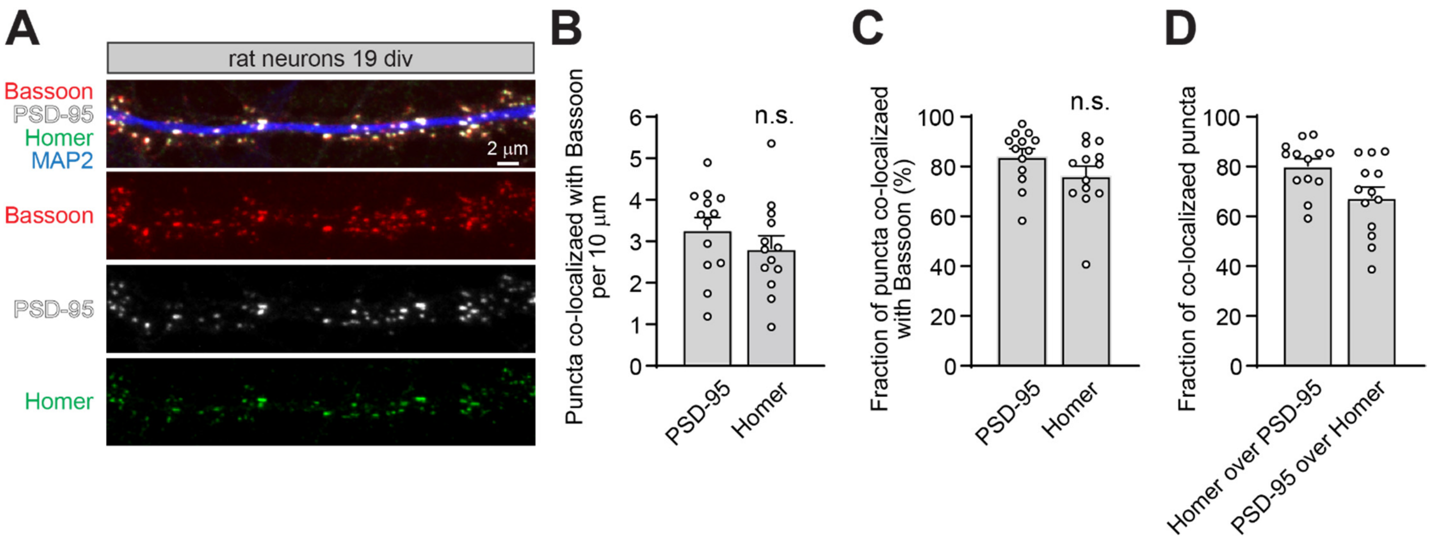

Supplemental Figure 4. Homer labeling of excitatory postsynaptic sites in cultured rat neurons

(A) Representative confocal images of rat hippocampal neurons at 19 div cultured in standard medium. Immunostaining for presynaptic Bassoon (red), the excitatory postsynaptic marker PSD-95 (shown white, acquired in far red), Homer 1 (green), and dendritic MAP2 (blue) is shown. Top, merged image. Bottom panels, individual channels.

(B) Quantification of images as in (A) showed that the density of synaptic PSD-95 puncta that colocalized with Bassoon per length dendrite was indistinguishable from the density of detected Homer puncta that co-localized with Bassoon.

(C) 84 ± 3.1% of postsynaptic PSD-95 puncta and 76 ± 3.8% of Homer puncta co-localized with Bassoon. The extent of their co-localization was indistinguishable.

(D) 80 ± 2.9% of Homer puncta co-localized with PSD-95 and 68 ± 4.3% of PSD-95 puncta co-localized with Homer.

(B-D, Student's t-test, two-tailed unpaired; n=13 neuron per condition, n.s., not significant)

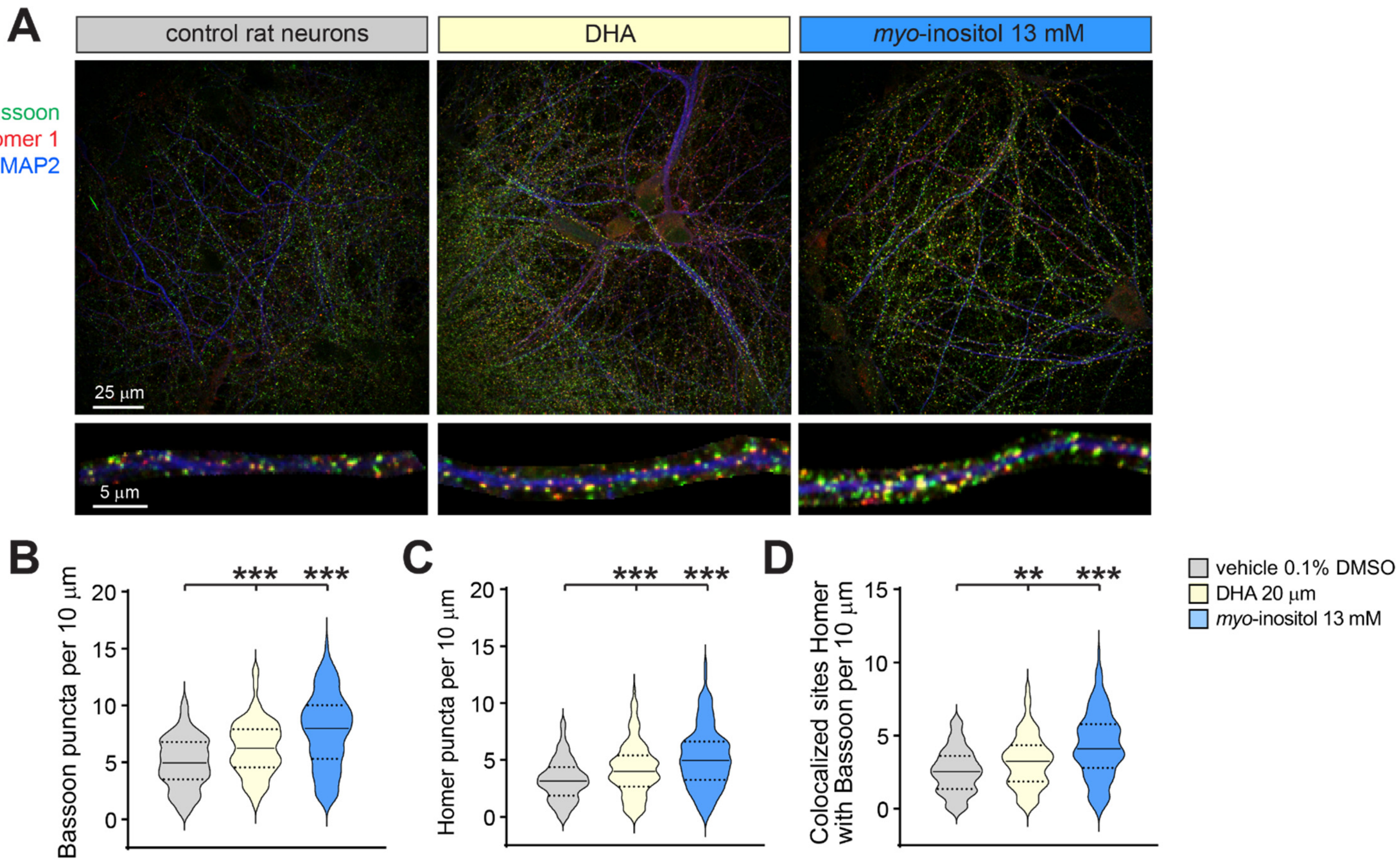

Supplemental Figure 5. *Myo*-inositol and DHA promote synaptic assembly in cultured rat neurons

(A) Representative confocal images show cultures of dissociated rat hippocampal neurons at 14 div treated with vehicle as control (left), 8 μM DHA (center), or *myo*-inositol at 13 mM (right). Immunostaining for presynaptic Bassoon (green), postsynaptic Homer 1 (red), and dendritic MAP2 (blue) was performed. Top, overview images. Bottom, enlarged dendritic segments.

(B-D) Quantification of images as in (A) determined that *myo*-inositol has bioactive roles in synapse development similar to DHA, with both compounds increasing the density of Bassoon-positive presynaptic puncta (B) and of postsynaptic Homer-positive specializations (C) per length of dendritic segment and of synaptic sites where these pre- and post-synaptic markers co-localize (D). The density of Bassoon puncta was increased in presence of *myo*-inositol by 57 ± 7 % compared to vehicle-treated control neurons, and the number of Homer-labeled sites was similarly elevated by 59 ± 7 %. *Myo*-inositol strongly promoted the number of postsynaptic Homer sites co-localized with Bassoon along dendrites by 66 ± 9 %. Violin plots show data distribution. Solid lines mark the median and dotted lines the quartiles. Asterisks show statistical differences of mean values, which are not plotted. (One-way ANOVA with Tukey's multiple comparison test; N=136 dendritic segments per condition, data from 6 independent experiments) ** $p<0.005$, *** $p<0.001$

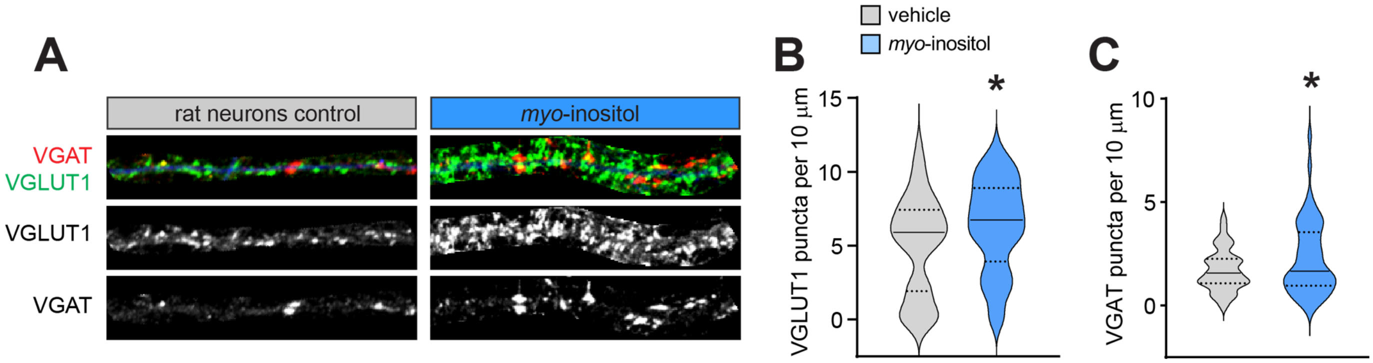

Supplemental Figure 6. Increased number of excitatory and inhibitory presynaptic sites in cultured rat neurons after *myo*-inositol treatment

(A) Confocal images of dendritic segments of rat hippocampal neurons treated with vehicle (left) or *myo*-inositol at 13 mM (right). Immunostaining for the excitatory presynaptic marker VGLUT1 (green) and the less abundant inhibitory presynaptic VGAT (red) was performed at 14 div. Top, merged images. Center and bottom, individual channels.

(B, C) Quantification of images as in (A) showed that *myo*-inositol supplementation increased the density of presynaptic sites positive for VGLUT1 (B) and VGAT (C). Violin plots show data distribution. Solid lines mark the median and dotted lines the quartiles. Asterisks show statistical differences of mean values, which are not plotted. (Student's *t*-test, two-tailed unpaired; N=54 dendritic segments per condition, data from 3 independent experiments) * $p<0.05$

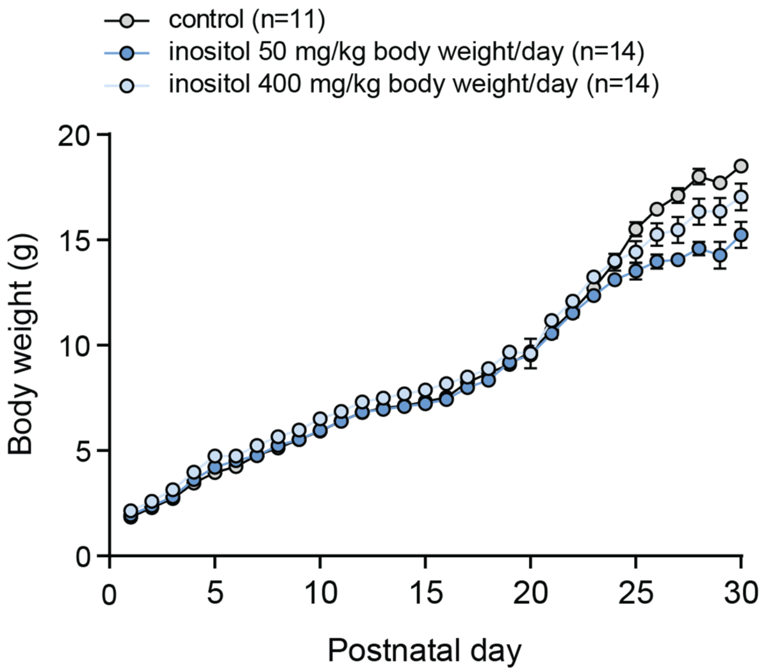

Supplemental Figure 7. Unaltered weight gain profile of *myo*-inositol vs. control-treated mice

Vehicle or *myo*-inositol were administered to mice orally daily from P1. Administration of inositol at 50 and 400 mg/kg body weight/day (dark and light blue, respectively) did not significantly alter normal weight gain compared to vehicle-treated animals (white). N=14 mice (*myo*-inositol at 50 and 400 mg/kg body weight/day), 11 (vehicle control).

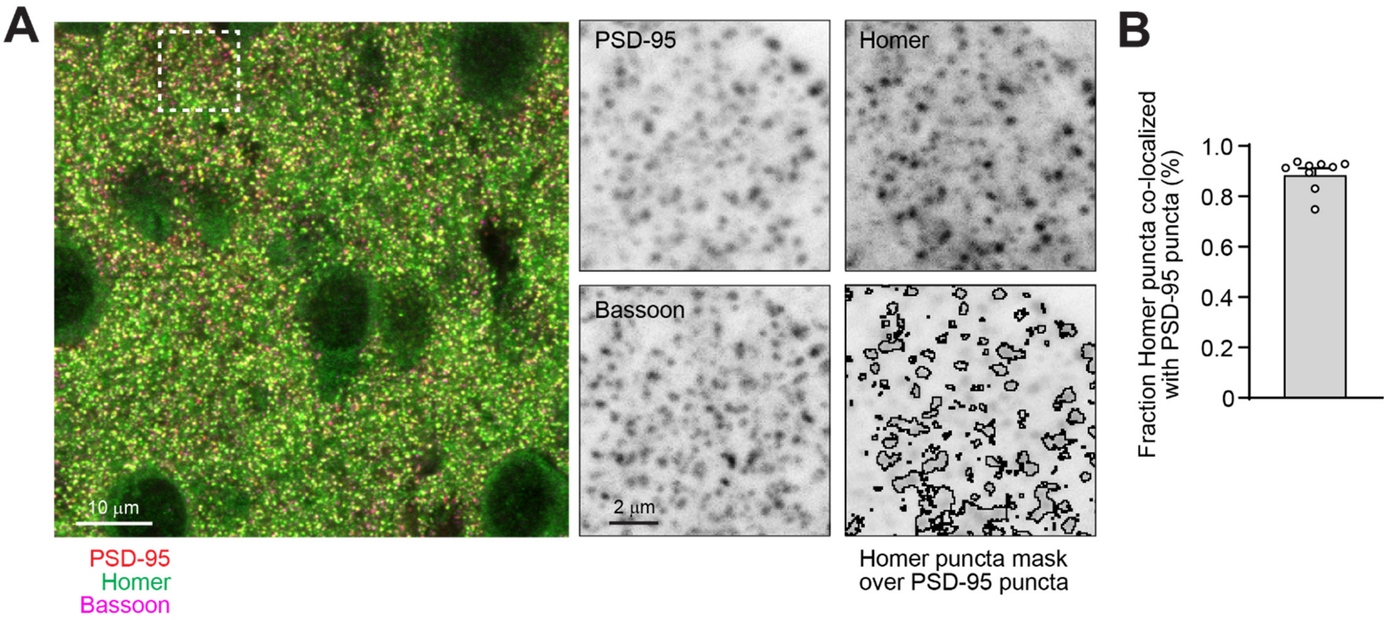

Supplemental Figure 8. Homer labeling of excitatory postsynaptic sites marked by PSD-95 in visual cortex

(A) Representative confocal image showing visual cortex V1 layer II/III of WT mice at P50 after immunostaining for postsynaptic PSD-95 (red), postsynaptic Homer 1 (green), and presynaptic Bassoon (magenta). Left, overview image with enlarged area marked by a box. Right, enlarged panels showing the three labeled channels and a mask of traced Homer puncta overlaid on the PSD-95 channel.

(B) Quantification of images as in (A) showed that 89 ± 2.1% of Homer puncta co-localized with PSD-95, consistent with localization to excitatory postsynaptic sites. The densities of Homer puncta at 52 ± 8.5 puncta per 100 μm^2 and of 60 ± 6.8 PSD-95 puncta per 100 μm^2 were indistinguishable (Student's t-test, two-tailed unpaired, p=0.49). A subset of 27% of PSD-95 puncta lacked apparent Homer staining. (n=9 sections, with 3 sections imaged for each 2 male mice and 1 female mouse and 4 ROIs quantified and averaged for each section)

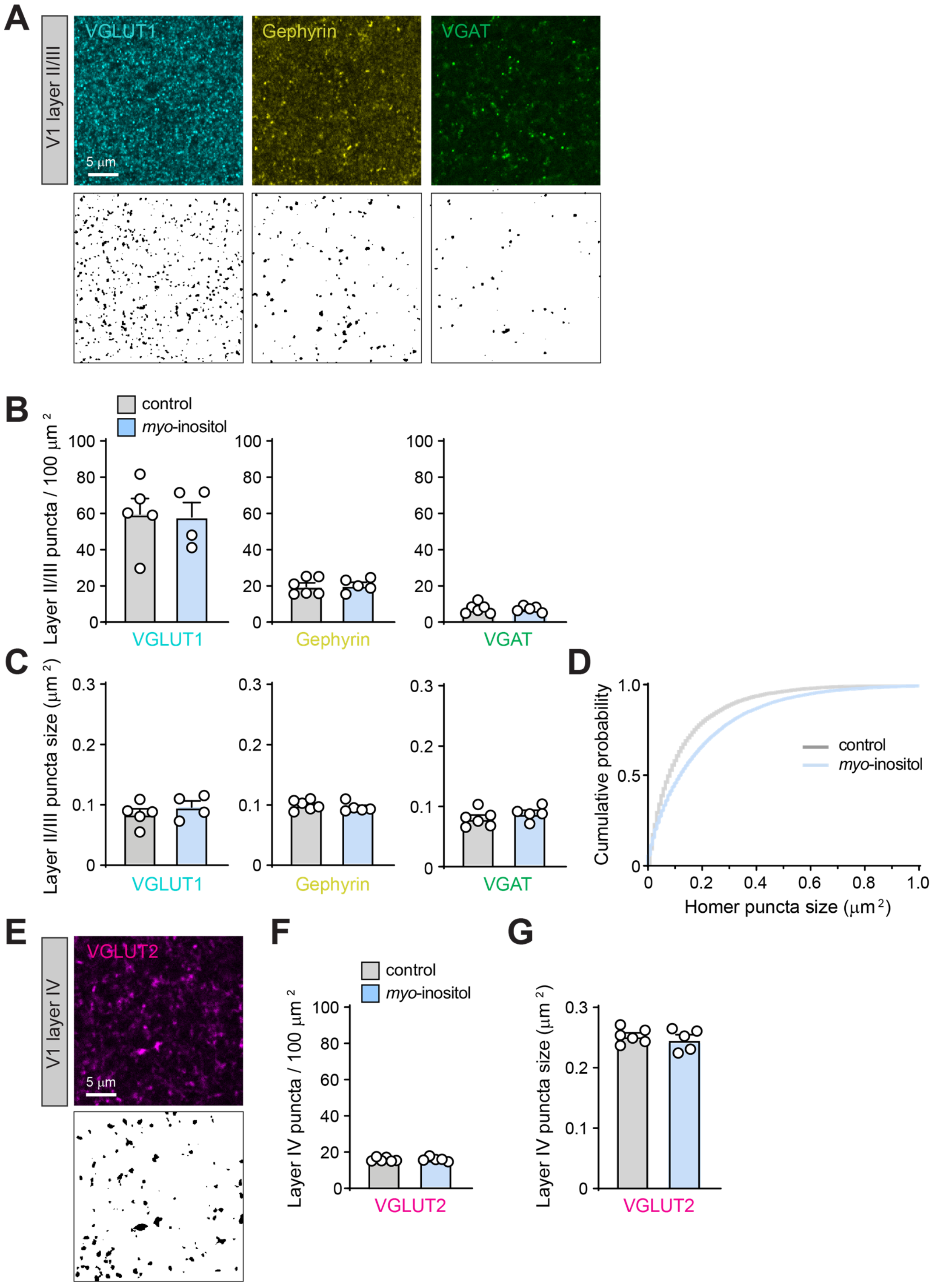

Supplemental Figure 9. Synaptic immunostainings in mouse V1 layers II/III and IV

(A) Top, representative synaptic immunostainings from control binocular primary visual cortex area V1b in mice at P35. Panels show single optical sections obtained by confocal microscopy in layer II/III after immunostaining for the excitatory presynaptic marker VGLUT1 and inhibitory post- and pre-synaptic markers Gephyrin and VGAT. Immunostaining for the excitatory postsynaptic marker Homer is shown in Figure 3B in the main text. Bottom, synaptic puncta masks after image processing.

(B, C) Quantification of synaptic puncta densities (B) and puncta sizes (C) from images as in (A) of control and *myo*-inositol complemented animals. No change in the size of VGLUT1 puncta was observed (N=5 control/4 *myo*-inositol complemented mice, *t*=0.897) and the size of puncta positive for the inhibitory pre- and post-synaptic markers VGAT (N=6/5, *t*=1.087, *p*=0.305) and Gephyrin (*N*=6/5, *t*=0.748) remained unchanged in layer II/III after *myo*-inositol complementation. Animal averages are shown, and means were compared. (Students t-test; Homer/VGLUT1, N=5 control mice with averages obtained from 3/2/2/2/2 sections per animal and N=4 *myo*-inositol complemented mice with 2/2/2/2 sections averaged per animal; VGLUT2/VGAT/Gephyrin, N=6 control mice with averages obtained from 1/1/2/2/1/2 sections per animal and *N*=5 *myo*-inositol complemented mice with averaged images from 1/2/1/2/1 sections per animal) * p<0.05

(D) Dietary *myo*-inositol complementation enlarges Homer puncta across all size populations. Cumulative distribution frequency plot for Homer puncta size in layer II/IIII shows a significant rightward shift towards larger sizes in *myo*-inositol complemented mice compared to controls.

(E) Immunostaining for the excitatory presynaptic marker VGLUT2 was obtained in layer IV. A representative immunostaining from a control mouse V1b at P35 is shown. Top, the panel shows a single optical section obtained by confocal microscopy in layer IV after immunostaining for VGLUT2. Bottom, VGLUT2 puncta mask after image processing.

(F, G) Quantification of presynaptic VGLUT2 puncta density (F) and puncta size (G) from images as in (E) of control and *myo*-inositol complemented animals. No change in the density (F) and size (G) was observed in layer IV. For statistical information, see the legend of panels, B, C.

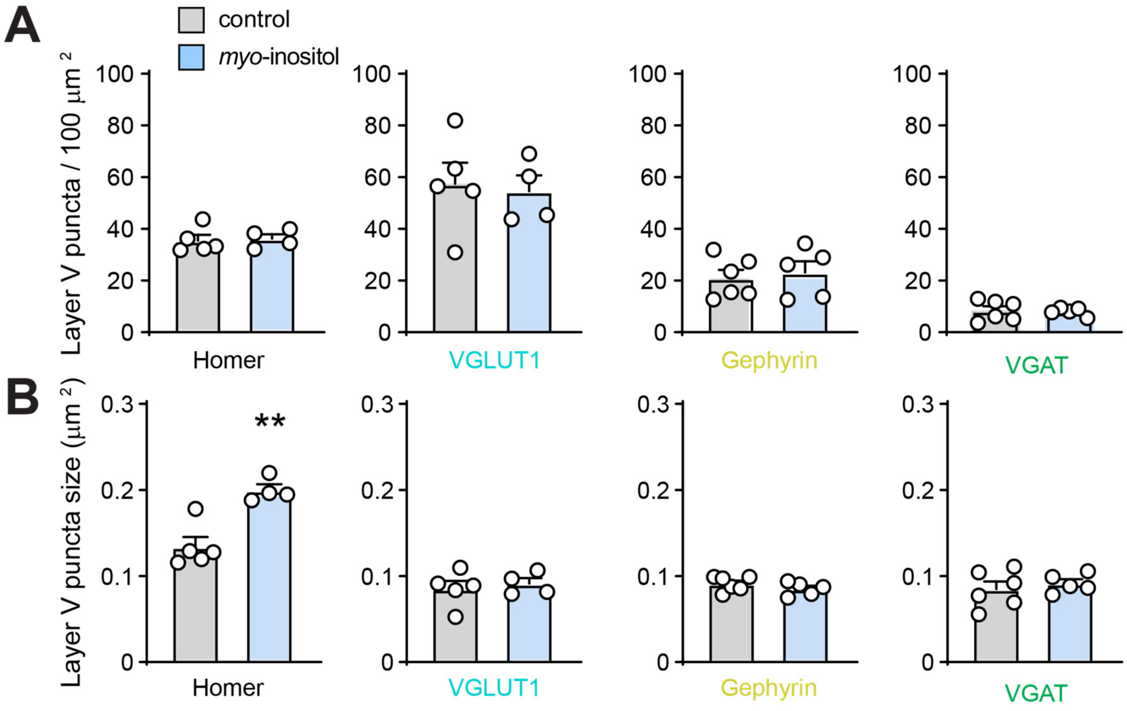

Supplemental Figure 10. Immunostainings for synapse markers in layer V of mouse V1

(A) Quantification of synaptic puncta densities of control and *myo*-inositol complemented mice in layer V of mouse binocular primary visual cortex area V1b at P35 showed no change in the indicated markers in control mice or mice receiving *myo*-inositol.

(B) Dietary supplementation with *myo*-inositol enlarged Homer puncta in layer V of mouse V1b at P35. No effect on the other analyzed synaptic markers was observed. (Student's *t*-test, two-tailed unpaired) ** $p<0.005$

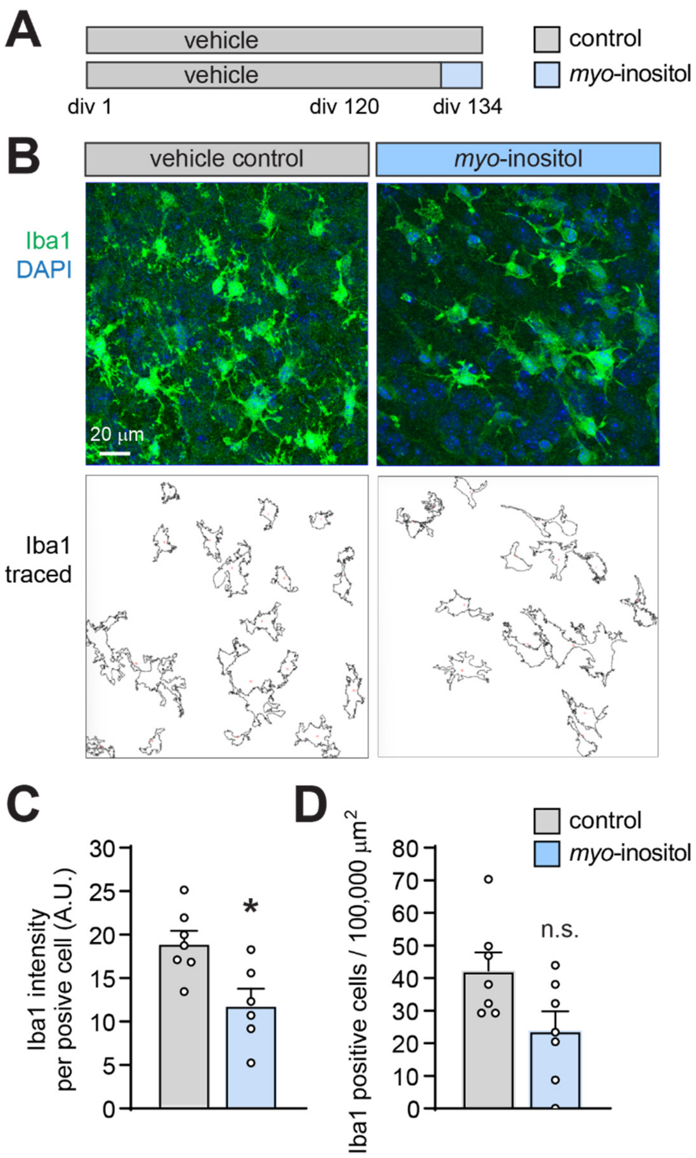

Supplemental Figure 11. *Myo*-inositol effects on Iba1 staining of microglia in organotypic slice cultures

(A) Organotypic slice cultures were prepared from the hippocampus of mouse pups and cultured for 134 days. A subset of cultured slices was treated from 120-134 div with *myo*-inositol at 1 mM.

(B) Top, representative confocal images of organotypic hippocampal slice cultures at 134 div cultured under control conditions (left) or including treatment with *myo*-inositol at 1 mM from 120-134 div (right). Slice cultures were stained for Iba1 to label microglia (green) and all cell bodies were labeled with DAPI (blue). Bottom, images after processing to trace Iba1-positive cells.

(C-D) Quantification of immunostainings as in (B) showed that *myo*-inositol treatment decreased the intensity of Iba1 immunostaining signal per cell (C) and caused a trend towards fewer detected Iba1 positive cells that was not significant (p=0.09). While the lifetime of microglia in organotypic slice cultures has to our knowledge not been determined, their lifetime in the mouse brain is 15 months (34) and the trend toward an apparently lower number of Iba1 positive cells upon *myo*-inositol treatment can be due to the reduction in Iba1 immunostaining signal, which can be expected to impede cell identification by the script, rather than due to fewer cells. For comparison of data in (D) with images in (B), note that the area shown in (B) is 185 x 185 μm^2 = 34,200 μm^2. (C-D, Student's *t*-test, two-tailed unpaired; N=7 control and N=6 *myo*-inositol slices prepared from 7 pups) * p<0.05, n.s., not significant.

SUPPLEMENTAL REFERENCES

1. P. R. Huttenlocher, Synaptic density in human frontal cortex - developmental changes and effects of aging. *Brain Res* **163**, 195-205 (1979).
2. P. R. Huttenlocher, A. S. Dabholkar, Regional differences in synaptogenesis in human cerebral cortex. *Journal of Comparative Neurology* **387**, 167-178 (1997).
3. P. Rakić, J. P. Bourgeois, M. F. Eckenhoff, N. Zecevic, P. S. Goldman-Rakic, Concurrent overproduction of synapses in diverse regions of the primate cerebral cortex. *Science* **232**, 232-235 (1986).
4. W. Gao *et al.*, Temporal and spatial development of axonal maturation and myelination of white matter in the developing brain. *AJNR Am J Neuroradiol* **30**, 290-296 (2009).
5. R. L. Stephens *et al.*, White matter development from birth to 6 years of age: A longitudinal study. *Cereb Cortex* **30**, 6152-6168 (2020).
6. P. Rakic, Mode of cell migration to the superficial layers of fetal monkey neocortex. *J Comp Neurol* **145**, 61-83 (1972).
7. P. Rakic, R. S. Cameron, H. Komuro, Recognition, adhesion, transmembrane signaling and cell motility in guided neuronal migration. *Current Opinion in Neurobiology* **4**, 63-69 (1994).
8. Z. Petanjek, I. Kostovic, M. Esclapez, Primate-specific origins and migration of cortical GABAergic neurons. *Front Neuroanat* **3**, 26 (2009).
9. J. C. Silbereis, S. Pochareddy, Y. Zhu, M. Li, N. Sestan, The cellular and molecular landscapes of the developing human central nervous system. *Neuron* **89**, 248-268 (2016).
10. G. Z. Tau, B. S. Peterson, Normal development of brain circuits. *Neuropsychopharmacology* **35**, 147-168 (2010).
11. R. T. Fremeau, Jr. *et al.*, The expression of vesicular glutamate transporters defines two classes of excitatory synapse. *Neuron* **31**, 247-260 (2001).
12. B. Xiao *et al.*, Homer regulates the association of group 1 metabotropic glutamate receptors with multivalent complexes of homer-related, synaptic proteins. *Neuron* **21**, 707-716 (1998).
13. J. E. Coleman *et al.*, Rapid structural remodeling of thalamocortical synapses parallels experience-dependent functional plasticity in mouse primary visual cortex. *J Neurosci* **30**, 9670-9682 (2010).
14. J. G. Woo *et al.*, Specific infant feeding practices do not consistently explain variation in anthropometry at age 1 year in urban United States, Mexico, and China cohorts. *J Nutr* **143**, 166-174 (2013).
15. K. A. Dingess *et al.*, Branched-chain fatty acid composition of human milk and the impact of maternal diet: the Global Exploration of Human Milk (GEHM) Study. *Am J Clin Nutr* **105**, 177-184 (2017).
16. J. G. Woo *et al.*, Longitudinal development of infant complementary diet diversity in 3 international cohorts. *J Pediatr* **167**, 969-974 e961 (2015).
17. T. E. Lipkie, A. L. Morrow, Z. E. Jouni, R. J. McMahon, M. G. Ferruzzi, Longitudinal survey of carotenoids in human milk from urban cohorts in China, Mexico, and the USA. *PLoS One* **10**, e0127729 (2015).
18. D. Ellingson *et al.*, Determination of free and total myo-inositol in infant formula and adult/pediatric nutritional formula by high- performance anion exchange chromatography with pulsed amperometric detection, including a novel total extraction using microwave-assisted acid hydrolysis and enzymatic treatment: first action 2012.12. *J AOAC Int* **96**, 1068-1072 (2013).
19. T. H. da Costa *et al.*, How much human milk do infants consume? Data from 12 countries using a standardized stable isotope methodology. *J Nutr* **140**, 2227-2232 (2010).
20. C. Bardy *et al.*, Neuronal medium that supports basic synaptic functions and activity of human neurons in vitro. *Proc Natl Acad Sci U S A* **112**, E2725-2734 (2015).
21. B. E. Carbone *et al.*, Synaptic connectivity and cortical maturation are promoted by the omega-3 fatty acid docosahexaenoic acid. *Cereb Cortex* **30**, 226-240 (2020).

22. T. Biederer, P. Scheiffele, Mixed-culture assays for analyzing neuronal synapse formation. *Nature Protocols* **2**, 670-676 (2007).
23. Y. Fukumoto *et al.*, Cost-effective gene transfection by DNA compaction at pH 4.0 using acidified, long shelf-life polyethylenimine. *Cytotechnology* **62**, 73-82 (2010).
24. K. Ichtchenko, T. Nguyen, T. C. Südhof, Structures, alternative splicing, and neurexin binding of multiple neuroligins. *J Biol Chem* **271**, 2676-2682 (1996).
25. K. Perez de Arce *et al.*, Topographic mapping of the synaptic cleft into adhesive nanodomains. *Neuron* **88**, 1165-1172 (2015).
26. B. E. Carbone, M. Iuliano, DendriteAnalysis16bit. GitHub. https://doi.org/10.5281/zenodo.7883923. Deposited 5/1/2023.
27. L. Stoppini, P. A. Buchs, D. Muller, A simple method for organotypic cultures of nervous tissue. *J Neurosci Methods* **37**, 173-182 (1991).
28. M. De Roo, A. Ribic, Analyzing structural plasticity of dendritic spines in organotypic slice culture. *Methods Mol Biol* **1538**, 277-289 (2017).
29. B. E. Carbone, M. Iuliano, SliceAnalysis. GitHub. https://doi.org/10.5281/zenodo.7865411. Deposited 4/25/2023.
30. J. Hoshiba, Method for hand-feeding mouse pups with nursing bottles. *Contemp Top Lab Anim Sci* **43**, 50-53 (2004).
31. S. M. Byun, R. Jenness, Estimation of free myo-inositol in milks of various species and its source in milk of rats (Rattus norvegicus). *J Dairy Sci* **65**, 531-536 (1982).
32. G. S. Tomassy, N. Morello, E. Calcagno, M. Giustetto, Developmental abnormalities of cortical interneurons precede symptoms onset in a mouse model of Rett syndrome. *J Neurochem* **131**, 115-127 (2014).
33. A. M. Winkler, M. A. Webster, D. Vidaurre, T. E. Nichols, S. M. Smith, Multi-level block permutation. *Neuroimage* **123**, 253-268 (2015).
34. P. Fuger *et al.*, Microglia turnover with aging and in an Alzheimer's model via long-term in vivo single-cell imaging. *Nat Neurosci* **20**, 1371-1376 (2017).
